# Biodegradable Natural Hydrogels for Tissue Engineering, Controlled Release, and Soil Remediation

**DOI:** 10.3390/polym16182599

**Published:** 2024-09-14

**Authors:** Ane Garcia-Garcia, Sara Muñana-González, Senentxu Lanceros-Mendez, Leire Ruiz-Rubio, Leyre Perez Alvarez, José Luis Vilas-Vilela

**Affiliations:** 1 Macromolecular Chemistry Group (LABQUIMAC), Physical Chemistry Department, Faculty of Science and Technology, University of the Basque Country UPV/EHU, 48940 Leioa, Spain; ane.garcia@bcmaterials.net (A.G.-G.); sara.munana@ehu.eus (S.M.-G.); leire.ruiz@ehu.es (L.R.-R.); joseluis.vilas@ehu.eus (J.L.V.-V.); 2 BCMaterials, Basque Center for Materials, Applications and Nanostructures, UPV/EHU Science Park, 48940 Leioa, Spain; senentxu.lanceros@bcmaterials.net; 3 Ikerbasque, Basque Foundation for Science, 48009 Bilbao, Spain

**Keywords:** hydrogels, biodegradable, tissue engineering, controlled release, soil remediation

## Abstract

This article provides insights into hydrogels of the most promising biodegradable natural polymers and their mechanisms of degradation, highlighting the different possibilities of controlling hydrogel degradation rates. Since biodegradable hydrogels can be designed as scaffolding materials to mimic the physical and biochemical properties of natural tissues, these hydrogels have found widespread application in the field of tissue engineering and controlled release. In the same manner, their potential as water reservoirs, macro- and microelement carriers, or matrixes for the selective adsorption of pollutants make them excellent candidates for sustainable soil amendment solutions. Accordingly, this article summarizes the recent advances in natural biodegradable hydrogels in the fields of tissue engineering, controlled release, and soil remediation, emphasizing the new opportunities that degradability and its tunability offer for the design and applicability of hydrogels.

## 1. Introduction

Hydrogels have emerged as a promising class of materials with diverse applications in very different fields, including biomedicine, cosmetics, drug delivery, tissue engineering and regenerative medicine, or agriculture and soil treatment [1,2,3,4,5]. This broad applicability is based on the capability of these materials, which are composed of crosslinked polymer networks, of absorbing and retaining large amounts of water, offering unique properties that make them highly attractive for the cited fields.

In recent years, significant progress has been made in the development of degradable hydrogels with enhanced biocompatibility, tunable mechanical properties, and controlled degradation kinetics. The ability of degradable hydrogels to undergo controlled degradation in response to specific stimuli, such as pH, temperature, enzymes, or light, presents opportunities for designing advanced materials with tailored properties for specific needs [6,7]. The design and synthesis of these hydrogels have been driven by the need for biocompatible materials with non-toxic degradation products that can mimic the extracellular matrix, support tissue regeneration, or provide the sustained release of therapeutic agents, nutrients, water, or agrochemicals [8]. By understanding the underlying principles governing the degradation mechanisms and properties of degradable hydrogels, researchers have been able to engineer materials with improved performance and functionality. In the design of degradable hydrogels, various factors need to be considered. These include the desired properties of the final product and the characteristics of the degradation byproducts, among others. Considering this, an important design choice is the election of the polymer or polymers that form the backbone of the hydrogel. Both synthetic and natural polymers have been broadly used for degradable hydrogel fabrication to great success. The former provide greater synthetic flexibility, control of physicochemical properties, as well as commercial availability, while the latter are preferred not only in terms of sustainability, but also for their higher biocompatibility, lower ecological impact, and innocuous degradation products (see Table 1).

In recent decades, the movement toward greener and more sustainable practices has also gained momentum, and much interest has been devoted to the use of naturally abundant feedstocks. It is considered as a promising path to reducing the great consumption of fossil resources and the consequent problems deriving from access to the limited reservoirs and the negative ecological impact caused by their extraction, processing, and lack of recyclability [9]. For this reason, biopolymers of natural origin have been highly researched for their potential applications in the synthesis of biocompatible and biodegradable hydrogels. Polysaccharides, such as cellulose, starch, alginate, chitosan, or hyaluronic acid, or polypeptides, like gelatin, have an inherent biodegradability due to the presence of cleavable bonds along the polymeric chain [10,11]. On top of this, the flexible polymer backbones and abundance of modifiable functional groups give rise to a wide variety of possible hydrogel morphologies and crosslinking strategies [12]. Thus, in this publication, we highlight the use of these natural biopolymers over their synthetic analogs for the fabrication of bio-friendly, degradable, functional materials.

The topic of degradable hydrogels has been approached from different angles in recent times. Some authors have provided insights into the various degradation mechanisms focusing on degradation efficiency and have discussed how degradability influences the design of hydrogel materials [7]. This interest in the degradation pathways and products arises following the concern caused by plastic waste accumulation. Other reviews center on the latest innovations in degradable hydrogels for specific applications. The field of biomedicine is where many of the current publications stem from, and one can find various examples of works dedicated to hydrogel materials for tissue engineering [13], sustainable bioelectronics [14], or drug delivery [15]. Some other studies focus on the physicochemical properties of hydrogels and the synthetic strategies to obtain them. Degradable hydrogels with bio-adhesive [16] or self-healing [17] properties, injectable [18], or stimuli-responsive [19] have also been reviewed separately.

This review aims to provide a comprehensive overview of the recent advances in the field of degradable natural hydrogels, providing an insight into how the different degradation mechanisms, properties of the polymers, and crosslinking strategies play a role in the design of hydrogel materials. By examining the current state of the art concerning degradable hydrogels, with a focus on the use of polymers from a natural origin, this work seeks to highlight the potential of these materials for tissue engineering, and their controlled release for biomedicine, ecosystem remediation, and sustainable agriculture. The simplicity of the formulations and the versatility of the most commonly used natural polymers for these applications of great interest and a promising future will be the focal point.

## 2. Degradation Mechanisms

Degradable materials are expected to maintain their properties for the desired lifetime and then degrade into smaller molecules that are ideally non-toxic and easily assimilated, metabolized, or removed. Degradation rates can be investigated using bulk property measurements, such as the monitoring of hydrogel swelling, mass loss, mechanical properties, solubilization, or the in vivo imaging and analysis of implanted materials. The hydrogel degradation rate also can be studied by monitoring direct bond cleavage or quantifying the concentration variation in degradation products [20].

Degradation mechanisms of hydrogels can be classified according to the topology or the bond breaking mechanism.

Looking at the general morphology of the hydrogels, the first classification of the degradation mechanisms could be differentiating between surface and bulk degradation. In surface erosion, degradation starts from the outermost layer of the material and the inner part does not degrade until the surrounding layers have been removed. Surface-eroding hydrogels decrease in thickness, losing material from the surface when the rate of degradation exceeds the rate of diffusion of solvents or reactive agents into the network, or where the catalyst is unable to penetrate the bulk material [21]. On the other hand, bulk degradation occurs simultaneously in various points of the material, regardless of the distance to the surface. However, these two mechanisms are not mutually exclusive, and most materials undergo both of them at different rates. Parameters such as the surface area, material density, bond breaking mechanism, and diffusion of molecules that catalyze or participate in the degradation process determine which of these topographic degradation mechanisms is dominant [22].

**Figure 1:**
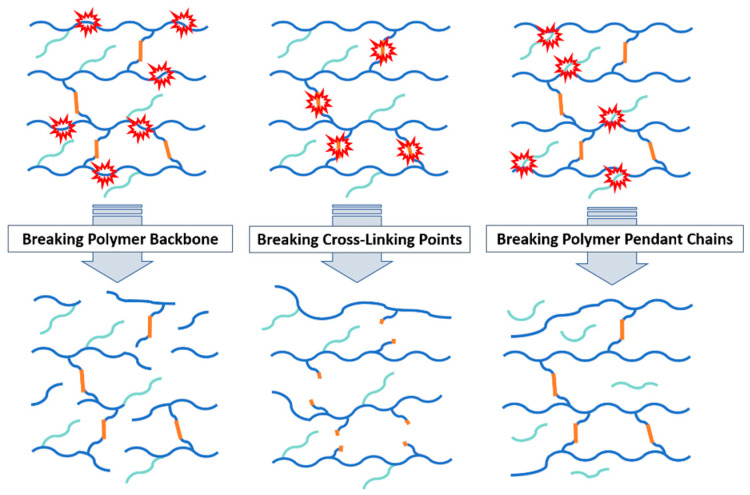
Degradation pathways of hydrogels based on possible bond cleavage points.

Another viewpoint to analyze hydrogel degradation is looking at the bond cleavage points of the gel network. For a crosslinked hydrogel structure, there are three possible breaking sites: the main polymer backbone, the crosslinking points, or the pendant polymer chains (Figure 1).

And finally, the degradation of hydrogels can be analyzed through the bond breaking mechanism (Figure 1). The most common bond breaking mechanisms for hydrogels are hydrolysis, solubilization/ionization, oxidation, photodegradation, and enzymatic degradation.

Due to their hydrophilic nature, hydrogels are susceptible to in vivo degradation by water. The process begins with the diffusion of water into the hydrogel matrix, causing its swelling and the disruption of secondary and tertiary structures that are stabilized by weak interactions (such as Van der Waals forces or hydrogen bonds), and ultimately solubilizes the material by the hydrolytic cleavage of the polymer or the crosslinker [7]. Natural polymers and macromolecules (e.g., polysaccharides, proteins, etc.) are generally biodegraded by hydrolysis followed by oxidation, either by the direct effect of the aqueous medium or catalyzed by enzymes [23]. Therefore, it is not surprising that biodegradable natural polymers, as well as their synthetic analogs, contain hydrolysable linkages, such as amide, ester, thioester, urethane, and glycosidic bonds, along the main polymeric chain. Many of these functional groups are also present in the crosslinking points, which also makes them susceptible to hydrolytic degradation [24]. The parameters that influence the hydrolytic degradation rate are those that affect the diffusion of water through the hydrogel matrix. Hydrogel pore size, pH, temperature, or the presence of H-bonding or repulsive interactions can favor or restrict water mobility and the availability of hydrolysable bonds. For example, the presence of negatively charged –COO^−^ groups in the polymer backbone has a repellant effect on the –OH groups that catalyze hydrolytic degradation, thus protecting the ester or amide linkages from –OH attack [25].

pH plays an important role in the degradation of hydrogels in an aqueous medium. Indeed, some degradation mechanisms are based on the pH-dependent water solubility of some polymers. When a change in pH causes the ionization (or protonation) of the functional groups of a water-insoluble polymer, it then becomes hydrophilic and solubilizes. Taking advantage of this concept, materials can be designed to regulate their degradation and the subsequent release of loaded molecules, based on the pH in the site of action by using polyacids and polybases. Hydroxypropyl methylcellulose phthalate, cellulose acetate phthalate, and polyvinyl acetate phthalate are some examples of degradable polymers with pH-triggered solubilization [26].

Enzyme-induced degradation involves a class of hydrolases that catalyze the cleavage of C-O, C-N, and C-C bonds, which is particularly important for the degradation of proteins and polysaccharides in living organisms [27]. Due to the highly specific binding of an enzyme and substrate, enzyme-induced degradation is sensitive to changes that affect enzyme conformation and activity, including pH, ionic strength, and temperature [28]. Degradation may begin by hydrolysis, but as the polymer breaks and the surface area and accessibility increase, enzymatic degradation may dominate. Therefore, biodegradation includes all types of degradation occurring in vivo, whether the degradation is due to hydrolysis or metabolic processes [26]. Since most enzyme-catalyzed reactions occur in aqueous media, the hydrophilic–hydrophobic character of synthetic polymers greatly affects their biodegradability. A polymer containing both hydrophobic and hydrophilic segments seems to have higher biodegradability than polymers containing either hydrophobic or hydrophilic structures only. In addition, the polymer chain must be flexible enough to fit into the active site of the enzyme [29].

**Figure 2:**
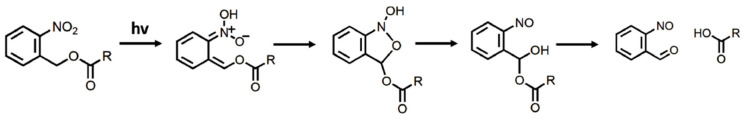
Photolytic cleavage mechanism of NB-based linkers.

Nowadays, there is rising interest in evolving approaches that allow external temporal and spatial control over the cleavage of crosslinks. Such control is possible by photodegradation by incorporating light-sensitive groups within polymer networks. Photolabile moieties are commonly integrated into the material design as linkers or pendant groups for modulating the structure, surface, mechanical properties, or matrix degradation. Nitrobenzyl (NB)-based linkers are some of the most commonly used photodegradable linkers. Their popularity can be attributed to their responsiveness to cytocompatible doses of light and the observed in vitro and in vivo biocompatibility of their cleavage products [30]. Under UV irradiation, an NB-photo labile bond is excited, resulting in intramolecular hydrogen abstraction followed by intramolecular rearrangement to produce a cyclic intermediate. The subsequent ring opening of the cyclic intermediate results in the formation of an o-nitrosobenzaldehyde and a carboxylic acid (Figure 2) [31]. The photolytic degradation rate of the hydrogel, as well as the synergy with other degradation methods, such as hydrolysis, are heavily influenced by the labile bond adjacent to the NB linker (different results for esters, amides, carbonates, and carbamates) [30].

In some cases, it is not possible to rely on an external trigger for degradation, such as a catalyst, heat, or light irradiation. Redox-induced degradation is caused by a change in the reductive or oxidative nature of the medium, in a similar manner to pH alterations initiating some hydrolytic degradation mechanisms. Redox-sensitive degradable hydrogels have been increasingly employed for innovative delivery systems for drugs and biomedicines due to their great stability in the circulation through extracellular fluids and rapid degradation in the reductive environment of the intracellular matrix [32].

**Figure 3:**
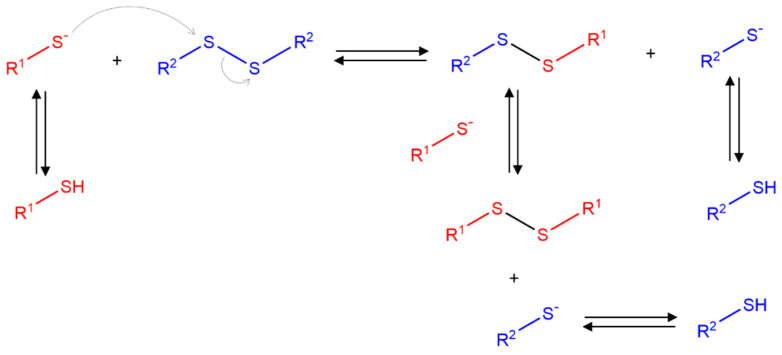
Set of reactions during disulfide–thiol interchange via SN_2_ substitution.

The design of redox-induced degradable hydrogels usually revolves around the incorporation of disulfide bonds, which are generally stable but quickly break under reductive conditions and experience exchange reactions in the presence of thiol or the thiolate anion. In biological environments, thiol-containing reducing agents, such as glutathione (GSH), are commonly found to perform a vital function in interchange reactions, like protein folding. In contrast to the low free thiol concentration in blood plasma, GSH is found at concentrations in the range of 1–10 mM in the cytoplasm. Furthermore, tumor cells typically exhibit an elevated production of GSH in the cytoplasm [33,34]. Thiols allow the easy and specific reduction in disulfide bonds. The reaction is essentially a disulfide exchange achieved by means of an SN_2_ nucleophilic substitution (Figure 3) [35].

Disulfide links are one example of dynamic covalent bonds. This type of chemical bond can undergo reversible reactions without an external energy source and makes the material self-healing, adaptive, or adhesive properties [36]. The reverse reactions can be triggered by changes in pH or temperature and the presence of reducing agents or competing molecules. Although these reversible reactions offer several advantages in hydrogel network formation and the resulting mechanical properties, their usability in degradation processes near physiological conditions is restricted. Dynamic covalent bonds can be degraded under certain stimuli, but the conditions are often not sufficiently mild (high temperatures or large amounts of acids or bases might be required) [37,38]. Therefore, two types of bond breaking should be considered when designing degradable dynamic bond-based hydrogels. First, degradation at the molecular level of the polymer chains (e.g., via hydrolytic or enzymatic degradation) that is independent of the reversible/dynamic bonds responsible for the self-healing and adhesive properties. Second, the loss of 3D structures by the cleavage of crosslinking points without the scission of the polymer backbones, in which the dynamic bonds are broken. This process is not considered a degradation, and therefore, the viability of hydrogels with dynamic bonds for biomedical or ecological applications depends on the non-toxicity and further degradability of the polymer backbones [17].

## 3. Degradable Bio-Based Polymers

**Figure 4:**
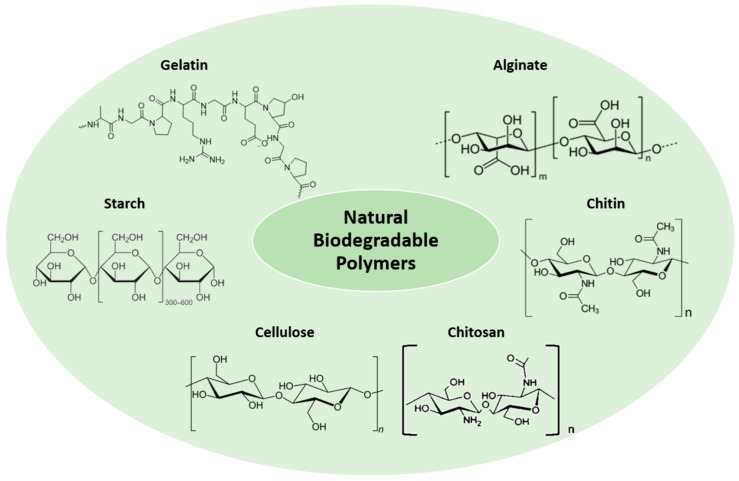
Natural biodegradable polymers for hydrogel synthesis.

As is described above, hydrogels formed from biodegradable polymers derived from natural sources are now replacing non-biodegradable hydrogels in many applications [39]. Accordingly, crosslinked natural polymers have been widely studied in recent years for their use in biodegradable hydrogels. It is believed that these naturally derived hydrogels have an advantage over other biomaterials in terms of degradation, due to their inherent biodegradability, and the reduced toxicity of their degradation byproducts [40]. Polysaccharides, such as starch, cellulose, chitosan, alginate, and polypeptides like gelatin, are the most common natural biodegradable polymers used as biodegradable hydrogels (Figure 4).

### 3.1. Starch

**Figure 5:**
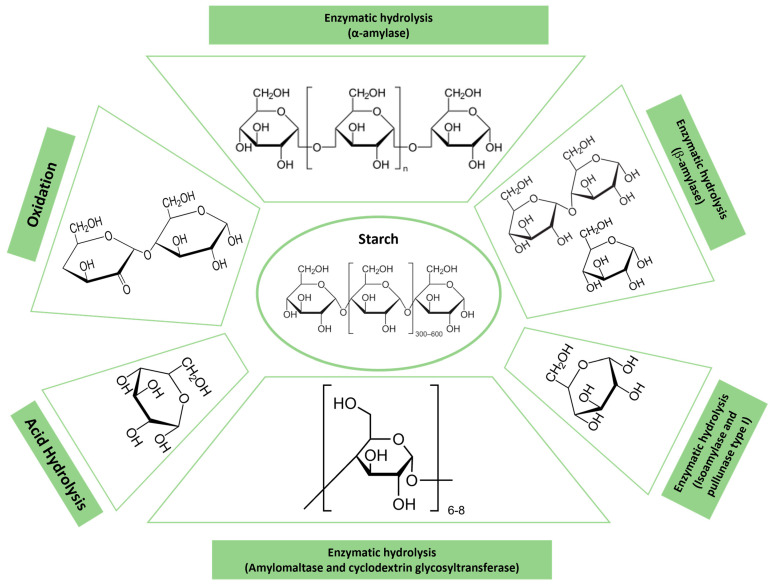
Schematic illustration of the degradation products of starch according to the corresponding degradation mechanisms.

Starch is a natural polysaccharide that is produced by plants and especially by crops (potatoes, corn, and rice) [41] in the form of granule. It is composed of a linear α-amylose structure (20–30%) and a branched amylopectin structure (70–80%) [42], linked through α-d-(1→4) and α-d-(1→6) glycosidic bonds [41]. Amylopectin is by far the most predominant polysaccharide type, and it primarily defines the properties of the starch. Starch is a versatile biomaterial due to its inherent biodegradability, abundance, cheapness, and non-toxic properties [41]. These materials are commonly used in food packaging [41], controlled release of fertilizers, water treatment, and the design of new drugs [42]. Therefore, the knowledge about its degradation is crucial to achieve controlled release or minimize the time required for the plastic to degrade in the environment [43]. There are several mechanisms by which starch degradation is initiated (see Figure 5), such as acid or enzymatic hydrolysis or oxidation [44]. Biologically, there are a variety of different enzymes involved in starch degradation. Four groups can be distinguished: (i) endoamylases (α-amylase), which are able to cleave α-(1→4) glycosidic bonds to form different oligosaccharides; (ii) exoamylases (β-amylase) that cleave both α-(1→4) and α-(1→6) glycosidic bonds to produce glucose or maltose; (iii) debranching enzymes (isoamylase and pullanase type I) that exclusively hydrolyze α-(1→6) glycosidic bonds; and (iv) transferases (amylomaltase and cyclodextrin glycosyltransferase), which cleave the α-(1→4) glycosidic bond to create a new glycosidic bond [45]. Conversely, starch is also cleaved by acid hydrolysis, but not by alkali hydrolysis, due to the stability of glycosidic bonds at a high pH. This process occurs in two phases: an initial rapid phase where the amorphous regions of starch are hydrolyzed, followed by a slower phase where both amorphous and crystalline regions are cleaved [46] resulting in a partial degradation of starch granules [47]. Additionally, starch undergoes degradation when exposed to oxidizing agents, such as periodate, dichromate, permanganate, persulfate, chlorite, and hydrogen peroxide, leading to depolymerization. This process results in lower viscosity dispersion, retardation in recrystallization due to the incorporation of carbonyl and carboxyl groups, and increase in the stability and transparency [48].

### 3.2. Cellulose

**Figure 6:**
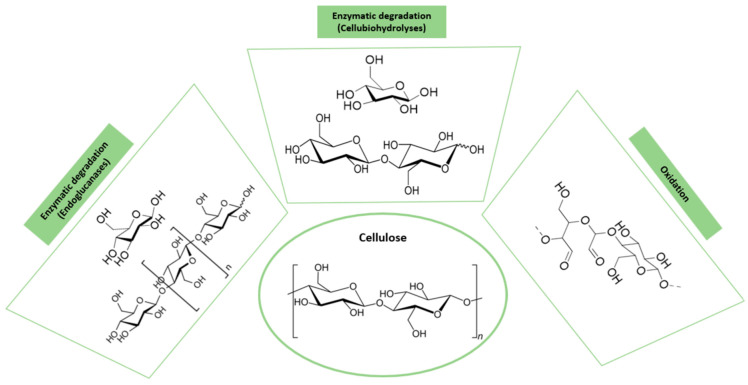
Schematic illustration of the degradation products of cellulose according to the corresponding degradation mechanisms.

Cellulose is a linear polysaccharide composed of repeating units of β-(1,4)-D-glucose. This polymer is the primary structural component of plant cell walls and is the most abundant polymer available in nature [49]. Cellulose has been widely used in healthcare and pharmaceutical industries due to its low cost, biodegradability, and biocompatibility [50]. Despite all its properties, cellulose presents a high infusibility and insolubility result and, therefore, chemical modification is required to enhance its processability [49]. The degradation of cellulose can occur through enzymatic-induced degradation or as a combination of several processes, such as hydrolysis, photodegradation, and oxidation, depending on the environment (see Figure 6). For instance, in nature, cellulose enzymatic degradation is carried out by various microorganisms and enzymes, secreted by cellulolytic bacteria and fungi [51] present in the air, water, and soil [52]. These enzymes, called cellulases, can be divided into endoglucanases, which hydrolyze β-(1,4)-glycosidic linkages, and cellobiohydrolyses, which react with the end groups of cellulose [53]. To claim final biodegradability, the complete conversion to CO_2_ or CH_4_ should be verified [54]. Oxidation is a promising method that creates new functional groups that facilitate its subsequent degradation. To this, potassium or sodium periodate, 2,2,6,6-tetramethylpiperidin-1-oxyl (TEMPO), phthalimide-N-oxy (PINO), perchloric acid, and hydrogen peroxide are the most common oxidizing agents, and primarily, carboxyl, aldehyde, and ketone are the resulting groups [55].

### 3.3. Chitosan and Chitin

**Figure 7:**
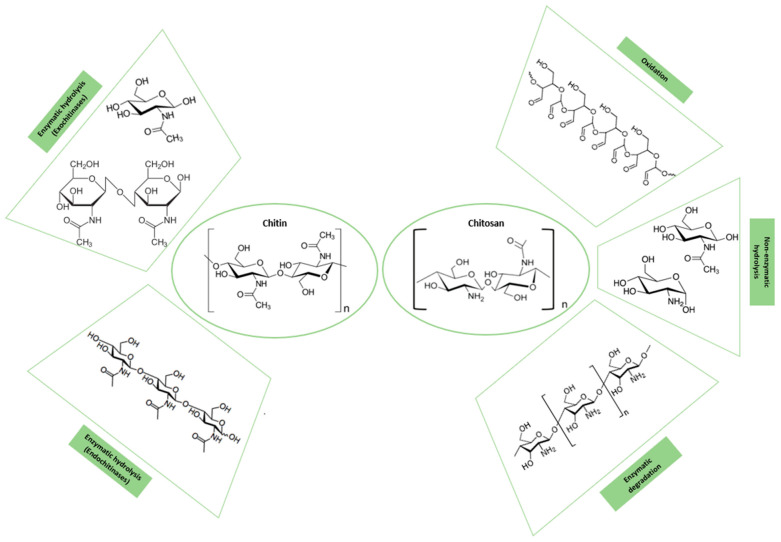
Schematic illustration of the degradation products of chitin and chitosan according to the corresponding degradation mechanisms.

Chitin, a polymer composed of β-(1→4)-linked N-acetyl-D-glucosamine, is the second most abundant organic resource in the world. It can be obtained from the exoskeleton of crustaceans and insects, and it is also present in plants, the cell walls of some fungi, and microorganisms [56]. Chitin is an interesting polymer due to its biocompatible, environmentally safe nature, and innate water solubility [57]. Therefore, this polymer is used in a wide range of applications, such as tissue engineering, wound dressing, drug delivery, and wastewater treatment [58]. Chitin is commonly degraded by an enzymatic hydrolysis called chitinolytic. This hydrolysis is carried out in the β-1,4-glycosidic bonds by chitinases, enzymes that are found in mammals, plants, insects, viruses, fungi, and bacteria [59]. Depending on the type of chitinase, exochitinases, or endochitinases, the degradation product varies (see Figure 7). Exochitinases cleave β-1,4-glycosidic bonds from the non-reducing ends of the chitin polymer, releasing disaccharide or monosaccharide units, whereas endochitinases cleave β-1,4-glycosidic bonds randomly at the internal sites of the polymer chain, yielding oligosaccharides of different lengths [60,61].

Chitosan, a linear and natural polysaccharide, can be obtained from chitin through the deacetylation reaction of N-acetyl-glucosamine [49]. Chitosan is a biodegradable polymer composed of randomly distributed units, N-acetyl glucosamine and D-glucosamine, bonded by with β-D-(1−4) glycoside linkages. Like other natural polysaccharides, chitosan possesses excellent biocompatibility [50], biodegradability, and nontoxicity [52]. Additionally, it exhibits properties such as antibacterial activity [49], antioxidant activity [7], immunostimulation [52], and tumor growth suppression capability [62]. It can also enhance both humoral and cell-mediated immune responses [63]. Considering these properties, this biopolymer and its derivatives are ideal candidates for applications such as tissue engineering [64], drug delivery [62], and agriculture, and soil treatment [65] in a variety of forms, such as nanoparticles, fibers, films, and hydrogels [66]. On the other hand, the applicability of chitosan is restricted by some parameters, including a specific molecular weight, and the degree of deacetylation, because they deeply influence its solubility, reactivity, cell response, and biodegradability. In terms of biodegradation, it is well known that the biodegradability of chitosan is highly dependent on its degree of deacetylation (DD); the higher the DD, the lower the degradation rate [67]. As many biopolymers, chitosan can be degraded by various mechanisms (see Figure 7). These degradation mechanisms include physical degradation processes [68]; chemical mechanisms such as oxidation, non-enzymatic hydrolysis, and enzymatic degradation through lysozymes [64]; and other enzymes present in the human body, such as chitotriosidase and acidic mammalian chitinase AMCase [69,70].

### 3.4. Alginate

**Figure 8:**
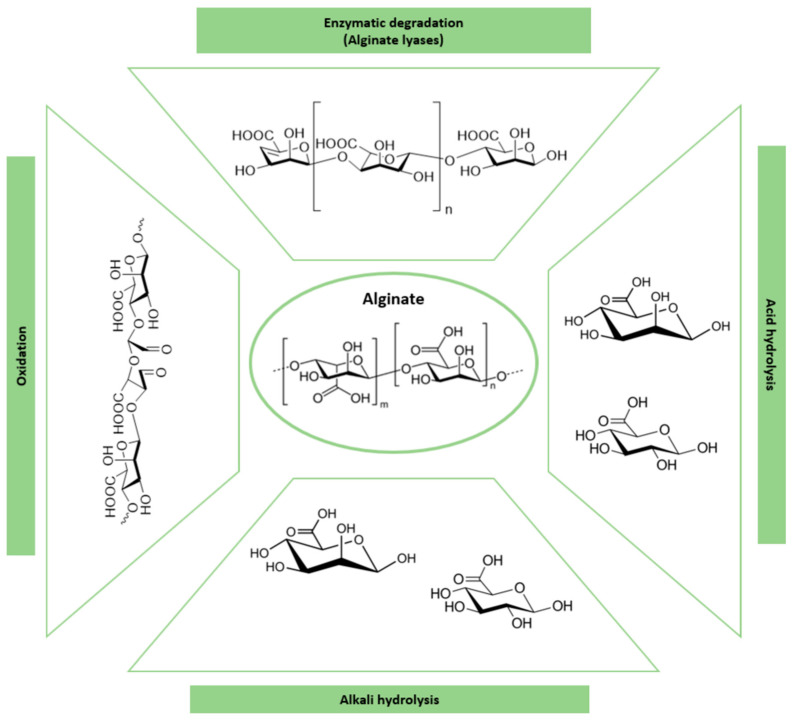
Schematic illustration of the degradation products of alginate according to the corresponding degradation mechanisms.

The monovalent form of alginic acid [71], alginate, is a hydrophilic [72] and linear polysaccharide composed of β-(1→4)-linked *D*-mannuronic acid and α-(1→4)-linked *L*-guluronic acid homopolymeric blocks. This well-known biomaterial is obtained from brown algae and depending on the algae source, blocks are randomly arranged or alternating [73]. Alginate is widely used for the controlled release of drugs and agrochemicals [74], water treatment, food industry, packaging, catalysts [75], and tissue engineering, due to its low toxicity, biocompatibility, and simple physical gelation in the presence of divalent cations such as Ca^2+^, Mg^2+^, Ba^2+^, and Sr^2+^ [76]. The alginate polymer is degraded by several mechanisms, including enzymatic biodegradation by alginate lyases, chemical hydrolysis (acid or alkali), and oxidation (see Figure 8). Alginate lyases, which can be isolated from various organisms, including algae, marine mollusks and some bacteria, viruses, and fungi, depolymerize alginate by the β-elimination of glycosidic bonds generating unsaturated oligosaccharides with C=C at the non-reducing end [77]. Alginate is also susceptible to hydrolysis in an acidic or alkaline medium, especially at elevated temperatures [78]. Above pH = 10.0, sodium alginate is degraded by the β-elimination mechanism [79], while at a low pH, alginic acid is formed due to acid catalyzed hydrolysis of glycosidic linkages [80]. Another attractive mechanism to degrade alginate includes partial oxidation, where sodium periodate oxidized alginate generating aldehyde groups in the structure. This mechanism does not interfere with its gel-forming capability, but depending on the degree of oxidation, molecular weight, pH, and temperature, the degradation rate may change [76].

### 3.5. Gelatin

**Figure 9:**
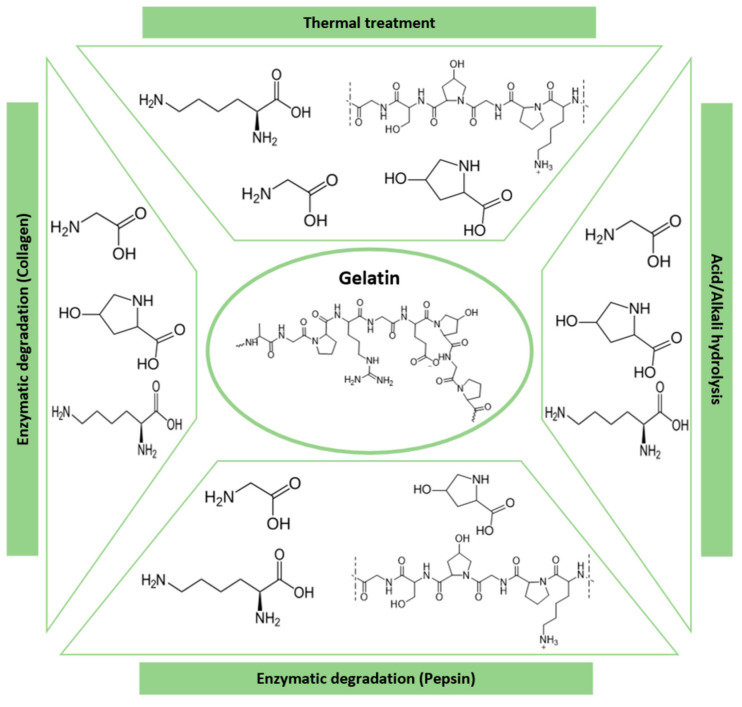
Schematic illustration of the degradation products of gelatin according to the corresponding degradation mechanisms.

Gelatin is a natural, soluble, functional protein compound derived from the hydrolysis of collagen, a fibrous protein found in bones, cartilages, tendons, and skin [52]. The chemical structure of gelatin consists of repetitive sequences of Gly-X-Y, where X is often proline (Pro) and Y is hydroxyproline (Hyp) [81]. Gelatin is a water-soluble, biodegradable, low-immunogenic, biocompatible, and easy to obtain polymer [50]. Consequently, it has been widely explored for the food industry, for example, to provide texture, foam, and clarity, as well as to stabilize food structure, for cosmetics and health products, pharmaceuticals, and for biomedical applications, including coatings, the preparation of biodegradable hydrogels [82], matrices for three-dimensional cell culture, and components of tissue engineering scaffolds [83]. Additionally, gelatin exhibits exceptional gel-forming capabilities in response to temperature changes [84]. However, the weakness and susceptibility to degradation in water of its hydrogels limits their applicability [50]. Gelatin degradation results in the loss of its gelling properties, and it can occur when exposed to heat, extreme pH conditions, enzymatic activity, etc. (see Figure 9). At low temperatures, chain mobility is reduced and inter/intrapolymeric hydrogen bonding is more stable, but when exposed to high temperatures, gelatin is denatured and, therefore, is more accessible for solvents and more susceptible to degradation via hydrolysis [85]. Regarding the pH, under acidic conditions, crosslinks between chains are primarily broken while peptide bonds are less frequently attacked. In contrast, at higher pH conditions, the more numerous peptide bonds are prone to breaking. On the other hand, in terms of enzymatic degradation, proteolytic enzymes, such as pepsin and pancreatin, are the main responsible enzymes. Pepsin is typically utilized in media with a pH below 6.8, while pancreatin is used for media with a pH of 6.8 or higher [86]. Furthermore, research related to gelatin degradation also mentions the use of collagenases. These enzymes are effective for degrading both gelatin and collagen and provide an additional option for the manipulation of gelatin-based biomaterials [87].

## 4. Applications

The physicochemical properties of hydrogels allow them to play an important role in a variety of applications. The crosslinked polymer chains act as a structural framework, and their hydrophilic nature and high swelling rate give rise to materials with a very high water content while still retaining the shape, generating small interfacial tension in aqueous environments. Those qualities provide physical similarities to body tissue, and combining this with biocompatible, biodegradable, and non-toxic polymers, it is possible to create materials capable of mimicking living tissue that are highly requested in the fields of biomedicine and tissue engineering [27]. Hydrogels are also ideal materials for the delivery of water-soluble nutrients and drugs, biomolecules, and even living cells due to their ability to provide the aqueous environment required for their preservation. Their water-holding capacity is also useful for controlling soil humidity and controlling the addition of fertilizers in agriculture. Additionally, hydrogels have been used for flexible electronics and wearable sensors because of their nice mechanical properties (being soft, flexible, and stretchable) and electrical conductivity [88].

However, conventional non-degradable hydrogels have some limitations for many of these applications. When hydrogels are implanted in patients for drug delivery or tissue regeneration, they must be removed from the organism after they have completed their purpose and are no longer necessary. Surgery is often required for their removal if the body is not capable of degrading, assimilating, or excreting the material, increasing the risk of additional complications and infections [89]. In agriculture and ecological applications, the presence of non-degradable hydrogels and polymers in soils and natural ecosystems can lead to additional contamination and bioaccumulation through the food chain [90]. Non-cleavable crosslinked networks have very poor recyclability, so their presence in sensors and actuators is also problematic for the environmental pollution problems caused by the generation of e-waste [91]. For these reasons, (bio)degradable hydrogels with non-toxic degradation products are highly demanded for biomedical and ecological applications. Next, in this review, we examine more closely the degradable hydrogel materials in the fields of tissue engineering and controlled delivery systems for biomedical and soil treatment fields. The focus is on the degradation capabilities required for each application, synthetic strategies to obtain functional materials that maintain the desired properties until the task they were designed for is completed, and how the degradation affects the physicochemical properties of the hydrogels (mechanical properties, crosslinking density, release rates, etc.).

### 4.1. Tissue Engineering Applications

Recently, the interdisciplinary field of tissue engineering, which merges materials science and cell biology, has brought new hope for addressing tissue repair challenges. The fundamental components of tissue engineering are the cells that create the tissue, scaffold materials that provide structural support for cells, and cytokines. Indeed, hydrogels are not mere 3D constructions required as the physical support of cells, but they must ensure the growth, proliferation, and expansion of cells and tissues [7]. Scaffolds must meet three basic requirements to guarantee the success of any tissue engineering approach. On the one hand, they must present high biocompatibility that guarantees a reduced risk of adverse immune responses. In addition, they must be degradable in order to enable temporary scaffolding and produce byproducts that, apart from not being toxic, must be easily metabolized or excreted by the body. Moreover, hydrogels must be able to mimic closely the physical and chemical properties of the natural extracellular matrix (ECM), providing the ideal environment for the transport of cells toward the specific site, stimulation of cell–polymer and cell–cell interactions, and the transportation of gases, nutrients, and waste [92].

Accordingly, natural hydrogels are rapidly evolving materials with significant implications for tissue engineering due to their mechanical and physicochemical properties, biocompatibility, their ability to degrade into non-toxic byproducts, and their capability to mimic natural tissues [93]. Thus, they have been widely used as scaffolding materials for tissue regeneration, cell encapsulation, wound dressing, drug and protein delivery, biosensors, or for preventing adhesion [94]. In recent years, these materials have extended the potential of tissue engineering creating more effective, personalized, and sustainable scaffolds. Bioprinted multicomponent systems that facilitate the creation of patient-specific tissues, in situ networks that provide a minimally invasive approach, or multi-responsive and biofunctionalizated hydrogels able to create complex microenvironments that mimic natural tissue are clear examples of the ongoing innovations in biodegradable hydrogels. These novel materials are promoting the success of effective strategies for in vitro and in vivo tissue regeneration.

In addition, hydrogels offer the possibility to be designed in order to support and enhance the regeneration process by means of supplementary strategies, such as the delivery of growth factor, drugs, and other bioactive molecules, enhancing tissue regeneration and the healing processes. Interestingly, the modification of the crosslinking density and composition of the hydrogels can be tuned to match the mechanical properties, the release kinetics, and the degradation profiles of hydrogels to the specific requirements of the different tissues, and even be gradually adapted to that of the variable nature of the growing tissue during the regeneration process. Specifically, the biodegradation of hydrogels must be adjusted to the formation of the new tissue. To achieve this, a balance is needed, because slow degradation can obstruct tissue function, while fast degradation can affect cell distribution, migration, and extracellular matrix synthesis [95].

Among all the natural polymers, polysaccharides, such as chitosan, alginate, and cellulose, and polypeptides like gelatin, are the most widely used polymers in the development of biodegradable hydrogels for tissue engineering. Since chitosan’s structure is very similar to glycosaminoglycans and their degradation products are molecules that typically take part in cartilage synthesis, chitosan scaffolds have been widely employed in cartilage tissue engineering [96]. Nevertheless, apart from cartilage, chitosan hydrogels have been employed over in recent decades as scaffolds for tissues with different properties, such as bone [97], skin [98], nerves [99], cardiac [100], or dental tissue [101]. In the case of this biopolymer derived from chitin, it is important to highlight that some important physicochemical properties, such as crystallinity and solubility, which mark its degradability, depend on the degree of the deacetylation (DD) of the polymer. Different studies with chitosan physical hydrogels have demonstrated that low acetylation degrees lead to a poor biodegradation ability. Tigh et al. [102] reported that scaffolds of chitosan hydrogels prepared by the freeze-drying method (2–3% *w*/*v*) exhibit higher interconnectivity, more biostability, and controlled degradation rates for high DD (>85% DD). These authors also confirmed the high biocompatibility and viability of scaffolds after 5 days of culture with an L929 mouse fibroblast. In this work, it is also concluded that higher DDs promoted better cell attachment and proliferation, and chitosan hydrogels were proved to be suitable for cell cultivation and superficial soft tissue engineering applications. Interestingly, the deacetylation grade and thus the degradation rate of chitosan hydrogels can be easily modulated. Rami et al. [95] varied specifically the DD of physical hydrogels of chitosan neutralized at basic pHs following a reacetylation procedure, including hydroalcoholic solubilization. These hydrogels were evaluated in vitro with human progenitor-derived endothelial cells (hPDECs) and human bone marrow mesenchymal stem cells (hBMSCs) and in vivo by subcutaneous implantation in female Wistars rats, and the great influence of the degradation kinetics on the cell invasion and tissue integration was shown. They demonstrated that low deacetylated hydrogels degrade quickly and, as a consequence, there is not enough time for cell invasion and ECM synthesis required to construct a neotissue. Highly deacetylated hydrogel systems (95% DD, 2.6% (*w*/*w*)), however, degrade slowly (60 days) and provide more elastic hydrogels, better cell adhesion on their surface, tissue regeneration, and restore tissue neo-vascularization. Additionally, the inflammatory response induced by these hydrogels remained low after 60 days of implantation in rats, which guarantees the long-term implantation in vivo. Therefore, these results make chitosan physical hydrogels excellent candidates with promising and innovative perspectives for applications such as tissue engineering and regenerative medicine.

The cited examples follow physical crosslinking methods, which, although they lead to poorer mechanical properties, are highly preferred to prepare stable hydrogels for tissue engineering applications due to their simplicity of fabrication and safe nature [92]. Certainly, since chitosan presents a high tendency for self-association, a compromised solubility from the balance of their hydrophobic forces, and a cationic nature in an acidic solution, this biodegradable polymer offers numerous possibilities to develop physically crosslinked hydrogels. Interestingly, despite the weaker nature of the physical crosslinking interactions in chitosan hydrogels prepared by neutralization, ionotropic gelation, freeze-drying, or thermo-sensitive gelation, their scaffolds induce favorable cell adhesion and proliferation toward human-skin fibroblasts [103], peripheral nerve cells [104], or even tough tissues, such as cartilage [105] and bone [106].

**Figure 10:**
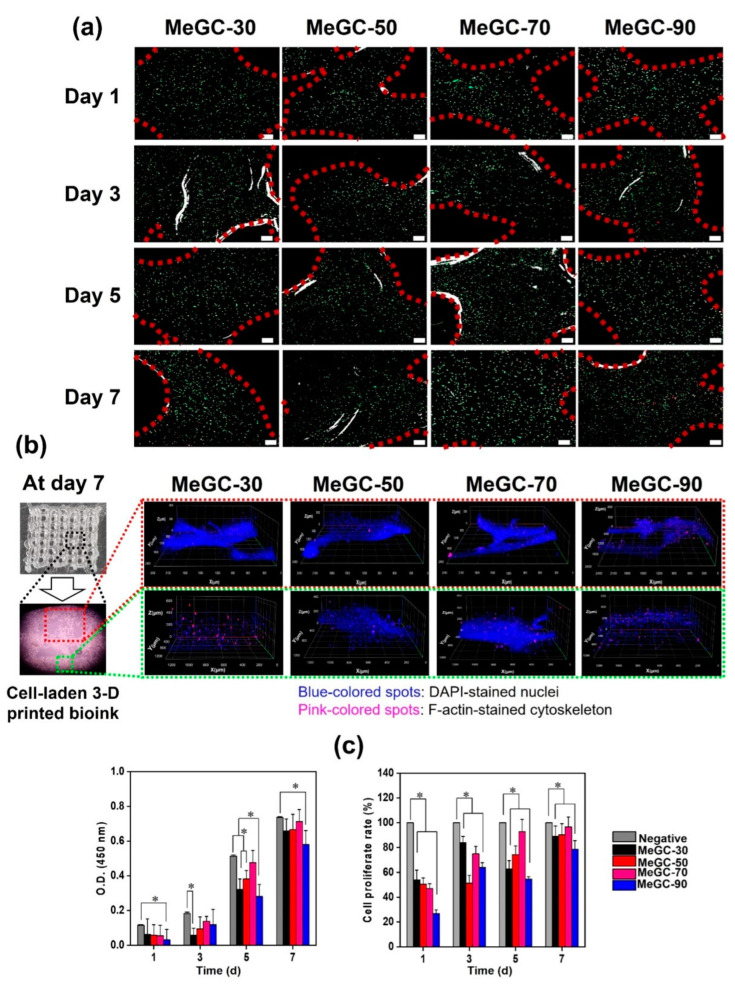
(**a**) Two-dimensional and (**b**) 3D live/dead fluorescence images of 3D-printed cells in MeGC-30, MeGC-50, MeGC-70, and MeGC-90 for 7 days of culture. Scale bars in (**a**) represent 200 μm. (**c**) Optical density and cell proliferation rate of printed MG-63 cells for 1, 3, 5, and 7 days (* *p* < 0.05). Reprinted permission from [108], Copyright (2022) Elsevier.

On the other hand, covalently crosslinked chitosan-based hydrogels, due to the strong and permanent interactions between the polymer chains, offer more control over physiological stability, higher degradation rates, and better mechanical strength, making them the most popular and effective option for tough tissues. Among the strategies recently followed in tissue engineering applications for the chemical crosslinking of chitosan, it is worth highlighting free radical photo-crosslinking. In 2012, Hu et al. [107] synthesized a visible-light crosslinkable chitosan hydrogel modified with glycidyl methacrylate employing different blue-light initiators. They observed that it was possible to control the degradation behavior of the scaffolds, reducing the penetration and accessibility of lysozyme by varying the initiator type and the irradiation time. These scaffolds enhanced articular chondrocytes’ growth and proliferation and presented an in vitro positive effect on osteochondral and chondral defects. These authors also confirmed that, after 21 days, the photo-crosslinkable hydrogels maintained a cell viability of 80% for different irradiation times. More recently, the photo-crosslinking of methacrylated chitosan has been exploited to develop 3D-printed hydrogels, which allows for a new perspective for the fabrication of engineered scaffolds. As an example, in 2022, Chang et al. [108] prepared a methacrylated glycol chitosan (MeGC) hydrogel to test the potential of the corresponding bioink, including modified chitosan, photoinitiator, MG-63 cells, and lysozyme, to develop 3D-printed scaffolds to be used for bone regeneration. Photo-curing enabled the accurate control of the physicochemical properties and degradation rates of the covalent networks by changing the photo-printing times. Three-dimensional-printed hydrogels experienced after 75 days differences in mass loss from 100 to 60% when the curing time was increased from 30 to 90 s. In addition to controlled degradability, 3D-printed hydrogels showed high protein adsorption, high cell viability (92%), cell proliferation (96%), and osteogenic capability in vitro after 7 days of culture (Figure 10).

The chemical reactions with crosslinking agents, such as aldehyde containing small molecules or the bio-safe genipin [109], have been broadly explored as an alternative to prepare scaffolds in tissue engineering. Chitosan–genipin hydrogels have been used to promote the re-epithelization effect, wound healing, and relieve inflammation and chronic infections [110].

Alginate hydrogels feature remarkable porosity, biocompatibility, biodegradability, non-toxicity, easy modification, and a chelating ability. Accordingly, it is one of the most promising polysaccharides for clinical translation and some alginate-based systems after approval by regulatory agencies are already available on the market. However, alginate presents some procedure difficulties, such as high viscosity and low solubility, and biological drawbacks, like poor cell adhesion properties and low degradability [111]. Owing to the absence of homologous enzymes in vivo, implanted alginate-based hydrogels are generally difficult to degrade and tend to maintain their original network, hindering cell infiltration and the formation of new tissue [112].

**Figure 11:**
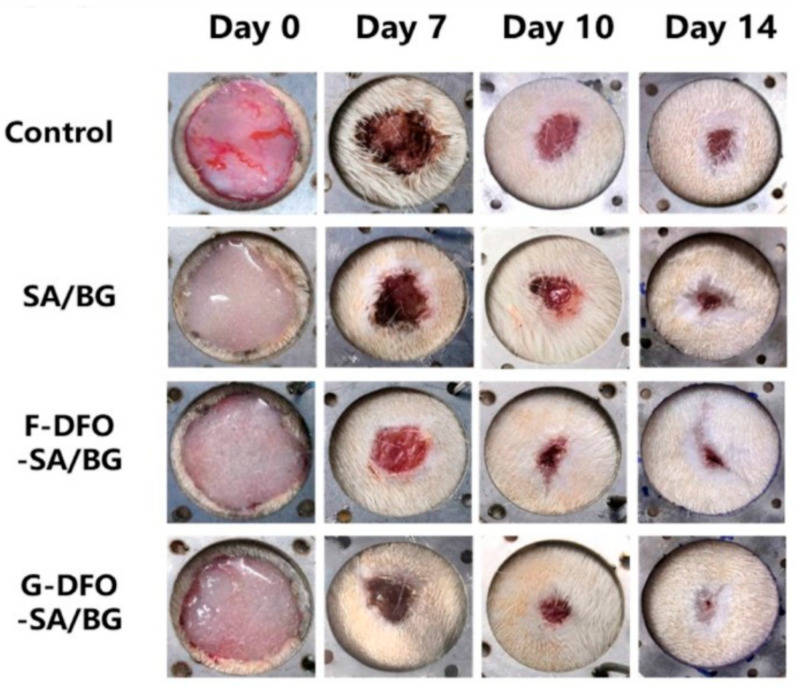
In vivo assessment of hydrogels for wound healing. Representative images of wounds treated with nothing (control), SA/BG hydrogel (SA/BG), physically encapsulated DFO-SA/BG hydrogel (F-DFO-SA/BG), and chemically grafted DFO-SA/BG hydrogel (G-DFO-SA/BG). Copyright © 2021. Zhang et al. [113].

Therefore, in recent years, many studies have been carried out to accelerate the degradation of alginate-based hydrogels. As aforementioned, alginate hydrogels are typically synthesized by the ionotropic gelation process induced by divalent cations. Since ionic bonds are stablished between calcium and the carboxyl groups of the guluronic acid units (G blocks) of alginate, the modulation of the number of carboxyl groups present in the G block has emerged as a strategy to control the degradation of alginate ionic networks. In 2021, Zhang et al. [113] followed this pathway by grafting deferoxamine (DFO) to the carboxyl groups of alginates, which led to the decrease in the crosslinking density, and, therefore, to an accelerated degradation in vitro and in vivo (see Figure 11). Additionally, they evaluated the biocompatibility, the angiogenesis function, and the wound healing ability of the scaffolds, and the results suggested that DFO-alginate-based hydrogels possess a great application potential in skin tissue engineering.

Another alternative strategy identified to accelerate the degradation of alginate hydrogels, is its oxidation by the reaction with oxidizing agents, such as sodium periodate. After oxidation, the obtained aldehyde functional groups are more susceptible to both hydrolysis and enzymatic degradation [114]. In the early 2000s, Bouhadir et al. [115], after investigating the in vitro degradation of ionically crosslinked partially oxidized alginate for cartilage tissue engineering applications, demonstrated that oxidation accelerates the degradation of alginate hydrogels, the degradation rate being highly influenced by the external pH and temperature. According to this study, a moderate oxidation grade (5%) allows the ionic crosslinking of alginate, and the corresponding hydrogels are able to be degraded after 9 days of in vitro incubation, while non-oxidized homologs did not degrade after months of incubation. In addition, their implantation into the dorsal region of SCID mice did not show an inflammatory response within 7 weeks, while new cartilage tissue formation was confirmed. Recently, in 2022, Wang et al. [76] exploited the ionic gelation of oxidized alginate as a 3D printing strategy. Three-dimensionally printed alginate scaffolds corroborated the significant increase in biodegradability derived from the oxidation of the biopolymer, showing full weight loss after 10 days of in vitro incubation, unlike non-oxidized alginate hydrogels. In addition, the in vitro cytocompatibility assay of oxidized systems with MC3T3-E1 cells did not show cytotoxicity after 5 days of incubation. In the literature, we can also find various studies combining ionic and photo crosslinking processes of partially oxidized alginate to obtain better control over degradation and allow the in situ formation of middle-term degradable scaffolds. In this context, Kandeloos et al. [116] engineered a biodegradable oxidized methacrylated alginate (OMA) hydrogel that, in the presence of CaCl_2_ and the adequate photoinitiator, led to a double crosslinked (ionic and photochemically) network. Methacrylation has been demonstrated to be an effective way to obtain better control of the degradability of alginate hydrogels. Additionally, in vitro cytocompatibility studies with L929 fibroblast cells demonstrated cell viability above 80%, indicating non-toxicity and good biocompatibility. Consequently, methacrylation is preferred for alginate covalent hydrogels as a versatile way to adjust mechanical properties without compromising their cytocompatibility. Similarly to other methacrylated polymers, several attempts have been made employing the methacrylated polymer for 3D photo-crosslinking. Sonaye et al. [117] reported the development of 3D-printed scaffolds by ionic and photo-crosslinking gelation to be used in muscle tissue engineering, however these authors, instead of oxidation, proposed the addition of gelatin to promote the biodegradation of the gels. Varying methacrylated alginate contents (4, 6, 8, and 12% *w*/*v*) were combined with gelatin (6% /v), and the resulting hydrogels showed a high modulation capacity of their degradation rate according to the multivariable nature of the composition of the scaffolds. Furthermore, the results obtained also showed, after 7 days of culture, an increase in cell growth the and high biocompatibility of the scaffolds.

In the case of cellulose, its highly ordered structure and the strong hydrogen bonding interactions between the glucose chains make cellulose highly resistant to biodegradation. However, cellulose degradation can be controlled by various crosslinking and chemical modification strategies that can be easily carried out thanks to the abundance of hydrophilic functional groups, such as hydroxyl, carboxyl, and aldehyde groups in its backbone [118]. As in the case of alginate, oxidation is one of the most exploited strategies. Chimpibul et al. [119] controlled the biodegradation of the oxidized cellulose scaffold for tissue engineering applications. They demonstrated that the Maillard reaction between the incorporated aldehyde and amino groups triggers cellulose degradation and, consequently, degradation is highly influenced by the degree of oxidation. In this case, an oxidation value of 43.3% led to total degradation after 45 min of study. Additionally, they studied the effect of the oxidation degree of cellulose scaffolds on the cell growth and morphology of cultured human bone marrow mesenchymal stem cells (MSCs). The obtained results showed that high oxidation degrees promote higher adhesion and cell growth. Finally, in vivo studies in which the scaffolds were implanted into the backs of rats showed low levels of inflammation, demonstrating that biodegradability and the in vivo response can be modulated through oxidation for subsequent use as materials in tissue engineering [119].

In recent years, in addition to chemical crosslinking strategies, countless studies have explored the combination of chemical and physical crosslinking to enhance the biodegradation, mechanical, and biological properties of cellulose. A clear example is the combination of cellulose with another natural polymer and the addition of a chemical crosslinker, such as epichlorohydrin (EPH). In this context, Mirtaghavi et al. [120] synthesized EPH-crosslinked cellulose nanofiber–gelatin scaffolds for tissue engineering applications. In this study, scaffolds were loaded with lysozyme for in vitro biodegradation, and the results showed that dual crosslinked hydrogels exhibited faster biodegradation after 56 days compared to hydrogels composed solely of cellulose. Additionally, cytocompatibility and cell proliferation assessments with human dermal fibroblasts (HDFBs) demonstrated enhanced biocompatibility, cell colony formation, and scaffold adhesion. An in vivo study on rats further confirmed excellent biocompatibility and an improved inflammatory response, making these scaffolds suitable candidates for tissue engineering applications [120].

**Figure 12:**
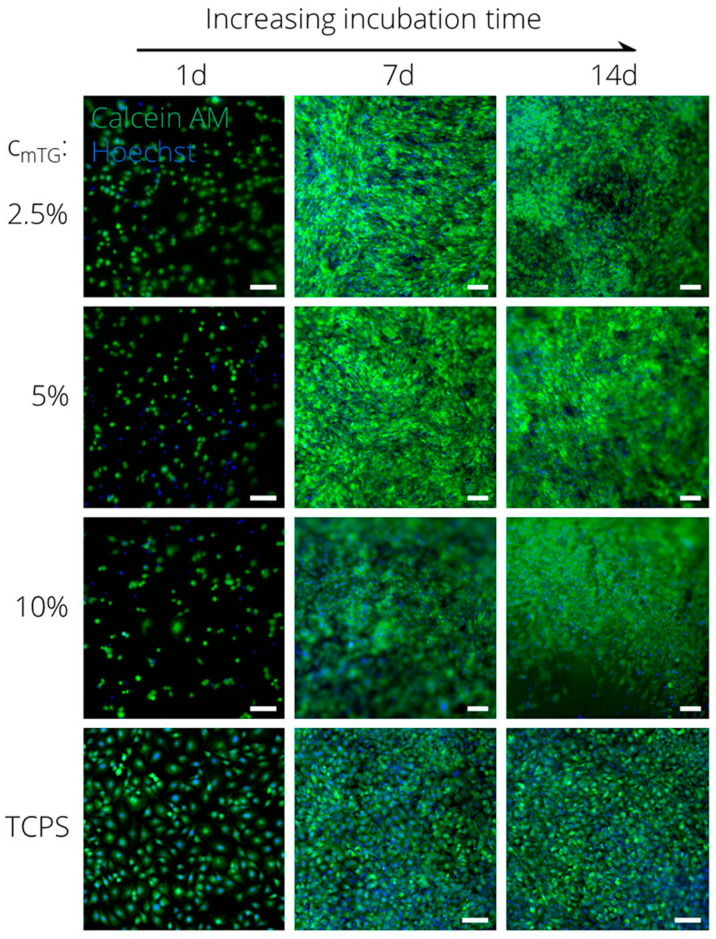
Proliferation study of ATDC-5 cells on ADA-GEL. Calcein AM/Hoechst stained ATDC-5 cells cultured on ADA-GEL crosslinked with 2.5%, 5%, and 10% (*w*/*v*) mTG for 1, 7, and 14 days (*n* = 3); scale bars = 100 μm. Adapted with permission from [122]. Copyright 2020, American Chemical Society.

Gelatin is another well-researched biomaterial used as a scaffold for applications in tissue engineering. However, as mentioned throughout this review, gelatin at high temperatures or in physiological conditions is denatured, leading to its redissolution and release from the scaffolds. Consequently, this biopolymer has limitations in terms of mechanical strength, and even its release can lead to hypoxia, compromising cell viability [121]. Therefore, many investigations have been devoted to applying chemical or physical crosslinking with the purpose of prolonging the degradation time and increasing the water resistance of gelatin. The introduction of alginate to gelatin networks is a clear example of this. When alginate and gelatin are crosslinked to form networks, typically both physical and chemical crosslinking methods are used together. Distler et al. [122] combined ionic and enzymatic methods to crosslink oxidized alginate and gelatin. In this article, the authors studied how the degradation and stiffness of the hydrogels could be modulated by varying the concentration of the enzymatic crosslinking agent (microbial transglutaminase (mTG)). The results reveal that it is possible to control the degradation behavior from fast (<7 days) to moderate (14 days) and slow (>30 days) by increasing mTG’s concentration (1–10% (*w*/*v*)). They also identified in vitro the adequate viability, attachment, and proliferation (see Figure 12) of NIH3-T3 and ATDC-5 cells, proving that these hydrogels are promising platforms for long-term cell culture investigations, such as cartilage, bone, and blood vessel engineering.

The recent study by Shehzad et al. [123] presents significant advancements in the development of 3D-printed gelatin-based hydrogels through dual crosslinking methods. The strategic integration of alginate and methacrylated gelatin has proven to be crucial not only for optimizing the structural stability and printing parameters of the gelatin, but also for regulating its degradation rate in relevant biological environments. Specifically, the data showed that the inclusion of alginate and methacrylated gelatin in the hydrogel matrix significantly improved the degradation profile of gelatin-based hydrogels after 14 days. The dual crosslinked hydrogel composed of 1% alginate, 4% gelatin, and 5% (*w*/*v*) gelatin methacrylate exhibited optimal properties in terms of the degradation rate, structural integrity, cytotoxicity, cell growth, and extracellular matrix deposition, which are essential properties for bone regeneration. Therefore, the combination of covalent and ionic crosslinking mechanisms into gelatin-based hydrogels not only enhances the material properties, but it also significantly improves cellular responses, including viability (around 100% after 14 days), proliferation, and osteogenic differentiation. These results underscore the potential of these hydrogels for applications in bone tissue engineering and regenerative medicine. A summary of recent works about degradable natural hydrogels for tissue engineering applications appears in Table 2.

In the past, with the aim to reduce immunological adverse reactions, hydrogels were intended to be passive materials. However, ideal biomaterials are evolving from passive to interactive, where hydrogels are able to adapt to the immunological responses and the physicochemical evolution of growing tissues promoting effective integration and regeneration. This emerging paradigm is driving the development of advanced stimuli-responsive hydrogels to be applied in tissue engineering [127]. These developments have led to the creation of scaffolds with unique properties that, by responding to different physical stimuli (e.g., temperature, light, electric, or magnetic fields) or chemical stimuli (e.g., pH, ionic factors, and chemical agents), enhance or support specific cell incubation, adhesion, differentiation, or proliferation activities [128]. For instance, Huang et al. [125] introduced Fe_3_O_4_ magnetic nanoparticles into gelatin hydrogels to produce smart scaffolds for cartilage tissue engineering. The authors examined the effects of a pulsed electromagnetic field on bone marrow-derived mesenchymal stem cells (BMSCs), and both in vitro and in vivo co-cultures of BMSCs within the gelatin-Fe_3_O_4_ hydrogel displayed high biocompatibility and complete knee articular cartilage regeneration, verifying that magnetically responsive hydrogels can promote cell proliferation and differentiation and, therefore, tissue regeneration.

These stimuli-responsive hydrogels have been also exploited in tissue engineering as in situ forming or injectable scaffolds, able to undergo a sol–gel transition as a consequence of an external stimulus, such as the pH, temperature, or ionic strength change, which allows them to become a gel once they are injected into the body. For instance, in 2020, Panita et al. [124] analyzed injectable hydrogels of chitosan in situ thermogelled with enhanced physical and mechanical properties by the addition of cellulose nanocrystals to be applied in bone tissue regeneration. Mouse pre-osteoblast cell viability and proliferation were observed after 7 days of culture, confirming the biocompatibility of the scaffolds. Additionally, the degradability and safety were also proved by an in vivo assay in mice, where after 14 and 30 days, the scaffolds demonstrated safe and significant biodegradation. These results suggest that this type of scaffold might be an excellent strategy to improve treatment for bone defects. Light has also emerged as a promising stimulus to develop injectable degradable hydrogels. In 2021, Ryoma et al. [126] developed a photosensitive scaffold using gelatin methacryloyl and riboflavin (RF) as a photoinitiator for bone regeneration. In this sense, these authors confirmed the great viability of KUSA-A1 cells and osteoblast differentiation after encapsulation in GelMA-RF hydrogels, verifying the potential application in bone tissue repair strategies.

### 4.2. Controlled Release Applications

Hydrogels have some intrinsic properties that make them suitable for encapsulation, transport, and the controlled release of smaller molecules. Their soft but insoluble nature, together with their high fluid retention capabilities and tunable bulk and surface properties, allow for the gradual release of the cargo and later degradation of the carrier, which is required for a variety of applications.

The most well-known applications of biodegradable hydrogels for controlled release are drug delivery systems. Hydrogels are excellent candidates for drug delivery because of their biocompatibility, bioinertness, and ability to preserve the activity of biomacromolecules. As we have seen for tissue engineering applications, hydrogels have similar properties to the extracellular matrix, allowing for a high acceptance of the hydrogel carriers in the body.

**Figure 13:**
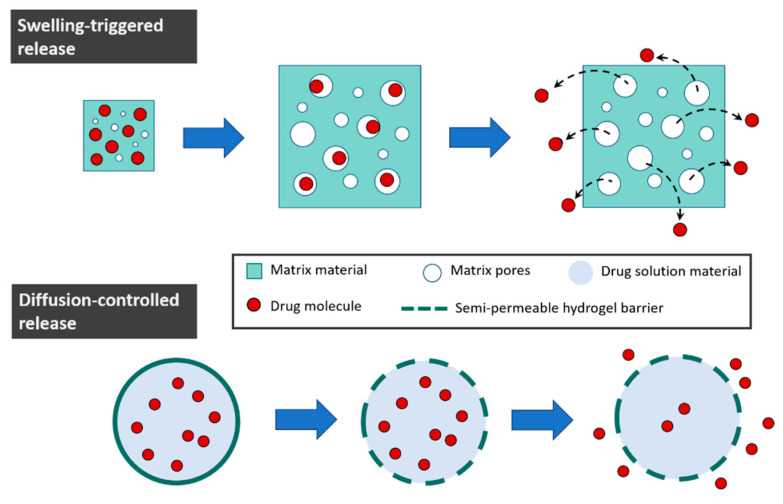
Controlled drug release mechanisms.

The mechanism by which the drug is released varies depending on the properties of the hydrogel matrix and the drug concentration. The main release routes are diffusion, swelling, and degradation of the carrier. In diffusion-controlled systems, whether the drug-loaded core is covered by a hydrogel film or the drug is uniformly distributed throughout the hydrogel matrix, the drug molecules are non-covalently entrapped in the material. In this case, the release of the drug is related to the pore size, tortuosity, and steric hinderance of the hydrogel network. Diffusivity is a function of drug concentration and, at the same time, drug diffusion from hydrogel matrix is also influenced by the degree of swelling and crosslinking density of the matrix. In a similar manner, swelling-triggered release is caused by a change in the pore size due to a volume expansion of the material (Figure 13). In these two cases, the release of the cargo is not necessarily tied to the degradation of the material and, even though swelling and diffusion can play a role in the degradation process, the liberation of the drug can still occur before degradation starts. In some cases, the swelling of the hydrogel is triggered by some external stimuli, such as pH or temperature changes.

Nevertheless, for biomedical and ecological applications, the degradation of the gel matrix is still desired, as breaking the material into smaller molecules would decrease the potential toxicity and facilitate the excretion, elimination, or assimilation of the material leftovers. In the cases when diffusion is heavily hindered or swelling is limited, the release mechanism is dependent on the degradation of the hydrogel. The degradation of the matrix can occur though surface erosion or bulk degradation of the polymer (as we have seen previously on this review) liberating the molecules trapped inside. However, drugs and proteins can be also incorporated in the hydrogel chemically tethered as pendant chains, so their release would be caused by the cleavage of these specific bonds, and not necessarily the complete degradation of the polymers. This approach was highly researched in 2010, when, for example, Brandl et al. [129] synthesized a biodegradable hydrogel by the step-growth polymerization of branched poly(ethylene glycol) with tethered fluorescently labeled bovine serum albumin (FITC-BSA) and lysozyme as model proteins that were slowly liberated upon hydrogel degradation.

An alternative to directly tethering the cargo protein or molecule to the polymer is the creation of an affinity-controlled system by incorporating network-bound ligands that form reversible binding interactions with the cargo. These moieties serve as an anchor for the therapeutic outcome and slow down the release rate [130]. This approach has commonly been used for heparin binding proteins [131,132] as well as other complementary binding partners, such as antibodies with antigens [133] or albumin with small-molecule therapeutics [134].

Having control over the degradation of the hydrogels is often crucial for controlling the liberation rate, especially when the cargo has a considerable size, such as when macromolecules, enzymes, or cells are encapsulated. Degradation can be triggered by a change in the medium (e.g., the carrier traveling through the body to areas with different pHs or temperatures), an external stimulus, or even by having a component that catalyzes the degradation entrapped in the hydrogel matrix. Campbell et al. (2018) [112] used an injectable alginate hydrogel for the controlled release of outgrowth endothelial cells (OECs) for the potential treatment of ischemic vascular diseases. The gel was loaded with the cells and alginate lyase, an enzyme that cleaves alginate polymer chains. Hydrogels incorporating 5 and 50 mU/mL of alginate lyase experienced approximately 28% and 57% losses of mass, respectively, and promoted up to nearly a 10-fold increase in OEC migration in vitro than nondegradable hydrogels over the course of a week.

**Figure 14:**
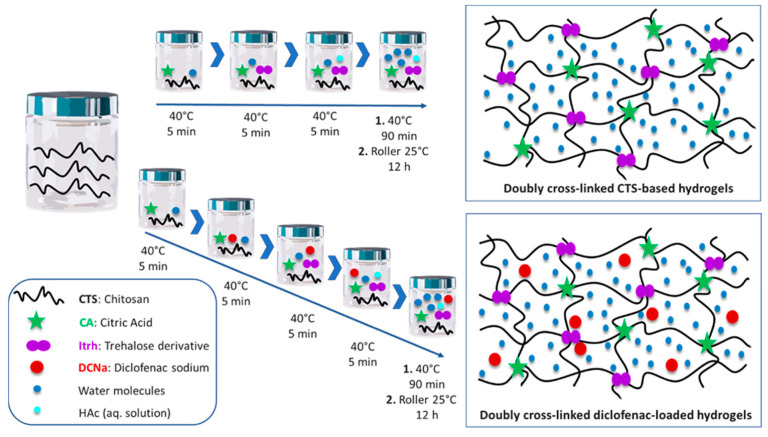
Schematic representation of the double crosslinked chitosan-based hydrogel for enhanced rheological properties and controlled release of sodium diclofenac. Citric acid acts as the ionic crosslinker and the trehalose derivative as the covalent crosslinker. Copyright © The Royal Society of Chemistry 2020, reproduced with permission from Iglesias et al. [136].

Regarding drug release, the reversible nature of ionically crosslinked networks is useful since, once the release of the cargo in the medium has been achieved, they can directly disintegrate into biocompatible components that will then be metabolized and eliminated from the body. However, they have weaker mechanical properties and are susceptible to easily losing the hydrogel structure through small changes in the medium, leading to sudden bursts instead of sustained release [135]. On the other hand, when chemical crosslinking is involved, a decrease in degradability is usually observed and labile bonds need to be introduced so that the gel can be broken under physiological conditions. Having a double crosslinked material can be a solution to combine the benefits of both physical and chemical crosslinking strategies. This approach can be applied by using polymers that can act as polyelectrolytes, such as chitosan. As an example, in 2020, Iglesias et al. [136] prepared a double crosslinked chitosan-based hydrogel to test the correlation between the variations in crosslinking and the liberation of the model drug sodium diclofenac and the mechanical properties of the material (Figure 14). The complete degradation of the hydrogel was achieved in 96 h. But both the rheological properties and the drug release profile changed according to the chitosan content and the crosslinking degree, with cumulative drug release varying from 17% to 40% for 72 h under physiological conditions. In this case, formulations with improved viscoelastic properties exhibited the lowest rates of drug release.

A more efficient delivery of the medication can be achieved through the evolution of drug delivery systems from passive materials to functional hydrogels capable of active delivery in the specific sites. These smart materials, which can respond to exogenous (externally applied) or endogenous (inherent to the organism) properties, improve drug efficacy and reduce side effects [137]. pH-responsive materials can be employed for selective drug administration in the acidic medium of tumor cells or the gastrointestinal system. A temperature-responsive hydrogel system allows the in situ formation of gel, which can be used to simply transport drugs to the target site and can be liquid or gel-like at environmental temperatures and change phases at elevated physiological temperatures in the body [138]. Light is a non-invasive and efficient external trigger, so photo-responsive hydrogels are often used for the delivery of imaging agents for diagnosis. Some hydrogels respond to the presence of specific enzymes or biomolecules and enable the release of the therapeutic agent. An example of this is real-time glucose-responsive carriers that are developed to facilitate the on-demand release of insulin. The physicochemical properties of these smart hydrogels are generally reversible to these applied stimuli [139], which allows for the loading and release of the cargo by simply changing the conditions of the medium. The inclusion of two or more responsive moieties within the polymers can create dual- or multi-responsive materials that can increase the level of precision of the treatment or be used in combined therapies, in which various therapeutic agents can be administrated in a single dose [140].

When designing a hydrogel for controlled release applications, apart from the release and degradation mechanisms and kinetics, one must also consider the morphology, size, and mechanical properties of the material. And, while the bulk hydrogel materials that are used for biomimetic scaffolding and wound dressing are often designed with the secondary purpose of slowly releasing drugs, nutrients or proteins that help in the regeneration process [27,141], or specific drug release applications, nano- and microscale materials are usually preferred. Combining the hydrophilicity, adaptability, and biocompatibility of hydrogels and the small size, high dispersibility, and long half-life in blood of the nanoparticles makes nanogels very promising materials for targeted drug delivery [142]. Drugs loaded within nanogels can easily pass through physiological barriers, thus increasing drug bioavailability. In addition, with the use of nanogels, less medication is required, and there are fewer doses per day, which reduces the toxicity of the medication [143].

For nanogel formulation for biomedical applications, some of the biopolymers are specially interesting due to their unique properties. A summary of some more recent examples of bio-based degradable hydrogels for drug delivery systems appears in Table 3, where the versatility of natural polymers for this application is shown through a collection of a variety of materials with different morphologies, cargos, and crosslinking strategies. In general terms, probably the two most-used natural polymers in drug delivery systems are the aforementioned chitosan (for its well-known electrolytic, antibacterial, cell-adhesion-promoting, antioxidant, and biocompatible properties) and hyaluronic acid, which has not been brought up yet in this review, but is widely used in the field of biomedicine. Hyaluronic acid is commonly used in targeted delivery systems for cancer treatment due to its unique tumor focusing ability, which works well in combination with other biopolymers. HA is a ligand for various cell surface receptors (CD44, LYVE-1, RHAMM, and HARE) that are overexpressed in tumor cells and nanogels preferentially accumulate in tumor tissues due to the enhanced permeability and retention (EPR) effect [144]. This conveniently complements the various stimuli-responsive degradation mechanisms HA nanogels can follow (redox reaction, photodegradation [145], thermodegradation [146], enzymatic degradation, etc.). For this reason, HA nanogels have been a popular option for the transport of bioimaging contrast agents [147,148,149] and antitumor drugs [150].

### 4.3. Soil Remediation and Sustainable Agriculture

**Figure 15:**
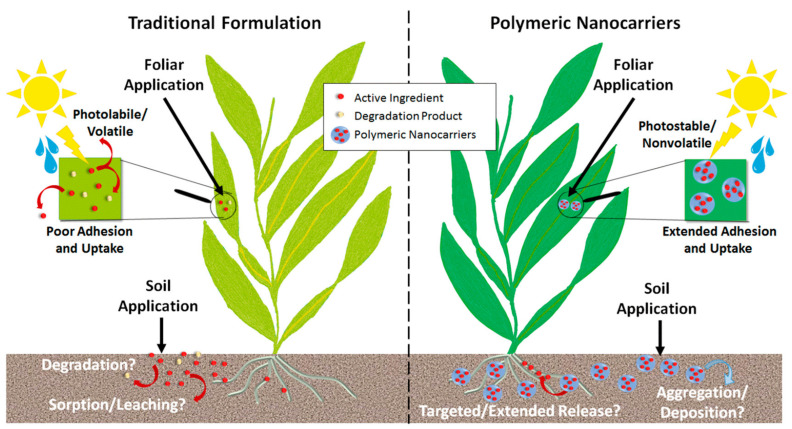
Applications of polymeric nanocarriers in the agricultural sector. Copyright © The Royal Society of Chemistry 2020, reproduced with permission from Shakiba et al. [158].

Moving away from biomedical applications, another field where hydrogels can play a promising role is soil and water remediation and sustainable agriculture. Correct C:N:P balance, humidity level, and the presence of micronutrients are essential to guarantee healthy soil conditions. In an effort to minimize the loss of artificially added nutrients to optimize agriculture and soil remediation strategies, the use of degradable hydrogels is contemplated for their sustained and controlled release. The polymer matrix provides protection from the influence of the environment and allows a slow diffusion of nutrients that would otherwise be lost through leaching or evaporation [155]. Similarly, hydrogels can also be applied for the controlled addition of fertilizers and pesticides in agriculture (Figure 15), thus reducing the amount of agrochemicals added to the soil, improving nutrient bioavailability and potentially reducing the risk of eutrophication and soil pollution [156]. Interestingly, the gradual release of water is also strongly valued in agriculture, because it allows for greater control over the moisture levels of the soil. Here, the high water-retention capacity of hydrogels proves to be a desired property. As a consequence of climate change, accentuated in recent decades, many regions in the world are facing the problem of desertification due to temperature variation, low rainfall, and increased droughts. The degradation of the soil and the loss of water due to evaporation and runoff negatively affect the uptake of water by plants and have significantly decreased the productivity of crops [157].

The initial use of biopolymers for the slow liberation of fertilizers was first reported in the 1980s by Otey et al., who used starch for controlled-release urea (starch-urea) [159]. It was not until 1990 that Teixeira et al. [160] introduced chitosan and alginate coatings. In 1996, Garcia et al. created lignin-based controlled-release coatings for urea fertilizers [161]. Apart from their biodegradability and non-toxicity, an advantage of using polymers of a natural origin for nutrient delivery is that their degradation products can act as an additional nutrient source. For example, alginate in soil degrades enzymatically or radiolytically, forming oligo-alginates that enhance germination, shoot elongation, and root growth [162].

The adjustable degradation of the carrier matrix is essential for agriculture, not only for the controlled release of agrochemicals, but also to prevent their bioaccumulation. Being able to fine-tune degradation helps us achieve the sustained release of various agrochemicals (e.g., pesticides and herbicides) over extended periods, thus reducing their excessive use and minimizing environmental pollution. Notably, glutathione (GSH), present in the cytoplasm of cells and in various plant tissues (e.g., root hair, wheat germ, and fruits) at concentrations ranging from 1 to 100 mg/g, provides a reducing environment that can trigger the redox degradation of hydrogels [163]. Additionally, conditions such as the anaerobic decomposition of organic material and lower soil pH also create reducing environments. Therefore, using disulfide and/or hydrazone-based crosslinking strategies to develop polymeric carriers is highly attractive for the efficient delivery of agrochemicals, as these carriers degrade in response to redox and/or pH stimuli [164]. Ankita Dhiman et al. [164] used this approach to create both pH- and redox-responsive alginate-based microgels for controlled herbicide release and simultaneous capturing of multiple heavy metal ions (Cu^2+^ and Hg^2+^). Monodisperse OAlgDP microgels were synthesized by using 3,3′-dithiopropionohydrazide as a crosslinker, which allows the incorporation of redox-responsive disulfide and pH-responsive hydrazone bonds in the polymer network. These microgels exhibited a prolonged release of diuron, lasting up to 380 h in the presence of 2 mM GSH and up to 240 h at pH 5. Moreover, they demonstrated very high efficiencies in capturing heavy metal ions from simulated soil leachate, with 98% efficiency for Cu^2+^ and 78% for Hg^2+^. Because of the larger scale of these applications, high water -olding capacity, lower synthesis costs, and mild degradation conditions are usually valued over the specific surface properties often needed for biomedical applications. For this reason, polymers of a natural origin and relatively simple crosslinking techniques are often found in the research. A collection of recent publications on biopolymer-based degradable hydrogels for soil is shown in Table 4, where the aforementioned trend of producing materials with a high water-retention capacity with mild synthesis and degradation conditions can be observed. In order to obtain the desired physicochemical properties, natural polymers are often combined with synthetic polymers, such as polyethylene glycol (PEG), polyacrylic acid (PAA), or polyvinyl alcohol (PVA). For example, Nandkishore Thombare’s team [165] created superabsorbent and moisture-retaining hydrogels based on acrylic acid-grafted guar gum crosslinking ethylene glycol with methacrylic acid (EGDMA). Warunee Tanan et al. [166] used semi-interpenetrating polymer network (semi-IPN) hydrogels of cassava starch-g-polyacrylic acid/natural rubber/polyvinyl alcohol blends (CSt-g-PAA/NR/PVA) following an aqueous solution polymerization method.

Other authors, such as R. G. Garduque et al. [167], opted for physical crosslinking through electrostatic interactions and H-bonding using sodium carboxymethyl cellulose sodium alginate and hydroxypropyl cellulose. The results show an accumulation of 1585% moisture and 8.38% fertilizer on a dry basis, with a decrease in fertilizer runoff by 28% and an increase in field capacity to 55% for an application of 5% hydrogel mass to slit soil. Biodegradability testing in soil revealed that, after 14 days, there was significant biodegradation of the hydrogel samples in soil. An important thing to notice was that, after the liberation of water, when the hydrogel was dried, the brittleness of the samples significantly increased, as the loss of moisture of the hydrogel resulted in an inability to support the internal polymeric structure [168]. To study the biodegradation of bulk-size hydrogel materials, a largely employed technique is soil burial. Soil burial is a very simple method that has the advantage of replicating real field conditions. However, this method can only be used for monitoring the overall degradation of the hydrogel, which is a combination of various degradation mechanisms, due to the complex composition of the soil matrix. The presence of water, pH, oxygen, enzymes, or microorganisms catalyzes different degradation processes. The measurement of the evolution of the mechanical properties of the hydrogel as well as the changes in the surface morphology (using, for example, microscopic imaging techniques, such as SEM or TEM) or chemical constitution (through spectroscopic or thermogravimetric analyses) can provide further information on the degradability of the hydrogels [20].

**Table 4:** Summary of some reported works about biodegradable natural hydrogels for agriculture and soil remediation.

Natural Polymer	Type of Hydrogel	Crosslinking Method	Application	Reference
Chitosan	Chitosan-based nanogels	Ionic crosslinking	Controlled release of nanofertilicers	[169]
Alginate	Alginate-based microgels	Covalent crosslinking	Controlled release of diuron herbicide	[164]
	Alginate, lignosulfonate, and konjaku flour-based hydrogels	Ionic crosslinking	Water and nutrient retention	[170]
Cellulose	Carboxymethyl cellulose-based nanogels	Reversible disulfide bonds	Controlled release of agrochemicals	[163]
	Carboxymethyl cellulose–alginate–hydroxypropyl cellulose-based hydrogels	Ionic crosslinking	Water storage and controlled nutrient release	[167]

## 5. Summary and Future Trends

Natural biodegradable hydrogels combine the unique properties of hydrogels, such as high aqueous solutions’ retention ability, biocompatibility, and resemblance to ECM, with the capability of being broken down into oligomers and low-molecular-weight materials easily and safely assimilated and/or eliminated by humans and living organisms. This combination, together with the increasing concern for environmental sustainability, make natural hydrogels ideal materials to be applied in both tissue engineering and soil remediation.

The remarkable functional properties of biodegradable hydrogels have already revolutionized the pharmaceuticals and biomedical fields, consolidating them as a competitive alternative to the existing non-degradable dosage and wound-healing approaches. Also, this revolutionizing effect has been extended to the tissue engineering field, in which biodegradable hydrogels are gaining increasing interest, replacing synthetic approaches. Biodegradable hydrogels offer great versatility in terms of their adaptability to the different kinds of tissues (from neural or vascular to bone or cartilage) and specific functionality (wound healing, injectable systems, ex vivo differentiation, and drug delivery). This versatility makes them a valuable tool for developing various therapeutic strategies. Future trends in the area include advanced fabrication techniques, like 3D bioprinting; enhancement of their functionality by the incorporation of bioactive molecules that guide the differentiation or regeneration process; or modification strategies that enable the responsive–adaptive behavior of the material to changing and complex environments.

Regarding the current environmental degradation of the soil derived from long-lasting droughts and intensive farming practices, biodegradable polymers have captured researchers’ interest as additives. The role of these hydrogels is not only to provide adequate water uptake, but also to act as temporary carriers of environmentally friendly fertilizers and/or nutrients and selective adsorbing systems of contaminants. The future trends of degradable hydrogels in soil remediation mainly focus on the incorporation of nano-technological solutions to enhance and control the properties of the hydrogels, the use of hydrogels responsive to external changes to promote targeted soil remediation, and the development of cost-effective and renewable materials that ensure their large-scale applicability.

Thus, although bulk, degradable, natural hydrogels present ideal properties to be successfully applied for tissue engineering, controlled release, soil remediation, and sustainable agriculture, the particularities of each application area make the difference in the design strategies and priorities of these fields. While tissue engineering highlights as its key priorities biocompatibility, mechanical properties, highly tailored degradation, and cellular response, new soil treatments focus on achieving high water retention, non-toxic byproducts, environmentally sustainable production, and cost-effectiveness. These differences in the goals and challenges establish differences regarding the development of hydrogels, and thus new synthetic approaches. Tissue engineering demands highly functionalizable, photo-crosslinkable, and stimuli-adaptative systems, in contrast to the need for raw biopolymers, and simplified formulations and procedures for applications in soil and agriculture.

Nevertheless, the control of the degradability of hydrogels is a key aspect that marks the effectiveness of any tissue engineering or soil amendment strategy. Regarding soil remediation treatments, it is well-known that rapid degradation negatively affects the soil remediation capacity of hydrogels, reducing their long-term water-retention and effective release or adsorption capabilities. The modulation of the biodegradation rate is also a key aspect in tissue engineering. Slow degradation processes may lead to the dysfunction of new tissues, while a high degradation rate prevents the adequate formation of ECMs, cell differentiation, and alters cell migration processes, among other negative consequences.

Consequently, a special interest has arisen in exploring the reaction mechanisms of the biodegradation processes of bio-based hydrogels, as well as the influence of the many physical parameters that alter their degradation kinetic profiles in order to achieve strict control of their degradability. Thus, knowledge is crucial to increase the significance of biodegradable hydrogels with respect to the synthetic ones in the ongoing research on tissue engineering and soil remediation. In this way, a special effort should still be made to achieve the goal of custom designing degradable hydrogels for advanced and targeted applications in the cited fields. Specifically, the translation of natural hydrogels from lab to clinical settings requires further investigations in terms of scale-up production, reproducibility, long-term stability, and regulatory compliance. Future research should focus on improving the strategies to achieve the controlled and sustained release of multiple active agents, adaptable and responsive-controlled degradation of hydrogel matrixes, and simplified formulations and synthetic procedures to reduce manufacturing complexities and possible harmful effects.

## Figures and Tables

**Table 1:** Comparison of natural and synthetic polymers for the fabrication of degradable hydrogels.

Characteristic	Natural Biopolymers	Synthetic Polymers
Biocompatibility	High	Polymer-dependent
Inherent biodegradability	High	Limited (polymer-dependent)
Degradation products	Non-toxic	Potentially harmful
Synthesis and modification flexibility	Medium	High
Tunable properties and kinetics	Medium	High
Working range (pH, T, and ionic strength, etc.)	Limited	Wide
Reproducibility	Likely	Controlled
Ecological impact	Low	Medium–high (polymer-dependent)

**Table 2:** Summary of some reported works about biodegradable natural hydrogels for tissue engineering.

Natural Polymer	Type of Hydrogel	Crosslinking Method	Tissue	Reference
Chitosan	Chitosan-based hydrogels	Physical crosslinking	Soft tissues	[102]
	Chitosan-based hydrogels	Physical crosslinking	Bone	[95]
	Chitosan-based hydrogel modified with glycidyl methacrylate	Chemical/Free-radical photo-crosslinking	Cartilage	[107]
	Methacrylated glycol chitosan hydrogels	Chemical/Free-radical photo-crosslinking	Bone	[108]
	Chitosan–genipin hydrogels	Chemical crosslinking	Skin	[110]
	Chitosan–cellulose nanocrystals-based hydrogels	Physical crosslinking	Bone	[124]
Alginate	Sodium alginate/bioglass hydrogel (SA/BG) grafted with deferoxamine (DFO)	Chemical crosslinking	Skin	[113]
	Oxidized alginate-based hydrogels	Physical crosslinking	Cartilage	[115]
	Oxidized alginate-based hydrogels	Physical crosslinking	General	[76]
	Oxidized methacrylated alginate (OMA) hydrogels	Physical and chemical crosslinking	General	[116]
	Alginate–gelatin-based hydrogels	Physical and chemical crosslinking	Skeletal muscle	[117]
Cellulose	Oxidized cellulose-based scaffolds	Chemical crosslinking	General	[119]
	Cellulose nanofibers-gelatin based scaffolds with epichlorohydrin	Physical and chemical crosslinking	General	[120]
Gelatin	Gelatin and oxidized alginate-based hydrogels	Physical and chemical crosslinking	Cartilage, bone, and blood vessels	[122]
	Methacrylated gelatin-alginate-based hydrogels	Physical and chemical crosslinking	Bone	[123]
	Gelatin with Fe_3_O_4_ magnetic nanoparticles	Chemical crosslinking	Cartilage	[125]
	Gelatin methacryloyl with riboflavin	Chemical crosslinking	Bone	[126]

**Table 3:** Summary of some reported works about biodegradable natural hydrogels for drug delivery.

Natural Polymer	Type of Hydrogel	Crosslinking Method	Delivered Drug	Reference
Chitosan	Chitosan-grafted-dihydrocaffeic acid-based hydrogel	Covalent crosslinking	Doxorubicin	[135]
	Chitosan-based double crosslinked hydrogel	Ionic and covalent crosslinking	Diclofenac sodium	[136]
Alginate	Alginate-carboxymethyl cellulose-based double-layer hydrogel	Ionic and covalent crosslinking	BSA and indomethacin	[151]
	Alginate-based nanogels	Covalent crosslinking	Caffeine	[152]
Cellulose	Cellulose–lignin-based hydrogels	Physical crosslinking	Paracetamol	[153]
Gelatin	Type-A gelatin and chitosan-based hydrogels	Ionic and thermal crosslinking	Tetracycline	[154]

## References

[1] MohanKumar B.S., Priyanka G., Rajalakshmi S., Sankar R., Sabreen T., Ravindran J. (2022). Hydrogels: Potential Aid in Tissue Engineering—A Review. Polym. Bull..

[2] Chamkouri H. (2021). A Review of Hydrogels, Their Properties and Applications in Medicine. Am. J. Biomed. Sci. Res..

[3] Vigata M., Meinert C., Hutmacher D.W., Bock N. (2020). Hydrogels as Drug Delivery Systems: A Review of Current Characterization and Evaluation Techniques. Pharmaceutics.

[4] Mitura S., Sionkowska A., Jaiswal A. (2020). Biopolymers for Hydrogels in Cosmetics: Review. J. Mater. Sci. Mater. Med..

[5] Wu Y., Li S., Chen G. (2023). Hydrogels as Water and Nutrient Reservoirs in Agricultural Soil: A Comprehensive Review of Classification, Performance, and Economic Advantages. Environ. Dev. Sustain..

[6] Kong H.J., Alsberg E., Kaigler D., Lee K.Y., Mooney D.J. (2004). Controlling Degradation of Hydrogels via the Size of Cross-Linked Junctions. Adv. Mater..

[7] Hosseinzadeh B., Ahmadi M. (2023). Degradable Hydrogels: Design Mechanisms and Versatile Applications. Mater. Today Sustain..

[8] Kaith B.S., Singh A., Sharma A.K., Sud D. (2021). Hydrogels: Synthesis, Classification, Properties and Potential Applications—A Brief Review. J. Polym. Environ..

[9] Bauer F., Nielsen T.D., Nilsson L.J., Palm E., Ericsson K., Fråne A., Cullen J. (2022). Plastics and Climate Change Breaking Carbon Lock-Ins through Three Mitigation Pathways. One Earth.

[10] Rajeswari S. (2017). Natural Polymers: A Recent Review. World J. Pharm. Pharm. Sci..

[11] Jain S., Jain A., Jain R., Chauhan N.S. (2024). Potential of Natural Polymeric Materials in Pharmaceutics. Pharmacol. Res.-Nat. Prod..

[12] Puertas-Bartolomé M., Mora-Boza A., García-Fernández L. (2021). Emerging Biofabrication Techniques: A Review on Natural Polymers for Biomedical Applications. Polymers.

[13] Yang J., Yu H., Wang L., Liu J., Liu X., Hong Y., Huang Y., Ren S. (2022). Advances in Adhesive Hydrogels for Tissue Engineering. Eur. Polym. J..

[14] Jia L., Li Y., Ren A., Xiang T., Zhou S. (2024). Degradable and Recyclable Hydrogels for Sustainable Bioelectronics. ACS Appl. Mater. Interfaces.

[15] Rizzo F., Kehr N.S. (2021). Recent Advances in Injectable Hydrogels for Controlled and Local Drug Delivery. Adv. Healthc. Mater..

[16] Xiong Y., Zhang X., Ma X., Wang W., Yan F., Zhao X., Chu X., Xu W., Sun C. (2021). A Review of the Properties and Applications of Bioadhesive Hydrogels. Polym. Chem..

[17] Grosjean M., Gangolphe L., Nottelet B. (2023). Degradable Self-Healable Networks for Use in Biomedical Applications. Adv. Funct. Mater..

[18] Li Y., Yang H.Y., Lee D.S. (2021). Advances in Biodegradable and Injectable Hydrogels for Biomedical Applications. J. Control. Release.

[19] Wang D., Duan J., Liu J., Yi H., Zhang Z., Song H., Li Y., Zhang K. (2023). Stimuli-Responsive Self-Degradable DNA Hydrogels: Design, Synthesis, and Applications. Adv. Healthc. Mater..

[20] Adjuik T.A., Nokes S.E., Montross M.D. (2023). Biodegradability of Bio-Based and Synthetic Hydrogels as Sustainable Soil Amendments: A Review. J. Appl. Polym. Sci..

[21] Laycock B., Nikolić M., Colwell J.M., Gauthier E., Halley P., Bottle S., George G. (2017). Lifetime Prediction of Biodegradable Polymers. Prog. Polym. Sci..

[22] Varde N.K., Pack D.W. (2004). Microspheres for Controlled Release Drug Delivery. Expert. Opin. Biol. Ther..

[23] Hahn S., Hennecke D. (2023). What Can We Learn from Biodegradation of Natural Polymers for Regulation?. Environ. Sci. Eur..

[24] Vroman I., Tighzert L. (2009). Biodegradable Polymers. Materials.

[25] Zhang H., Zhou L., Zhang W. (2014). Control of Scaffold Degradation in Tissue Engineering: A Review. Tissue Eng. Part. B Rev..

[26] Singh B., Sharma N. (2008). Mechanistic Implications of Plastic Degradation. Polym. Degrad. Stab..

[27] Hu B., Gao J., Lu Y., Wang Y. (2023). Applications of Degradable Hydrogels in Novel Approaches to Disease Treatment and New Modes of Drug Delivery. Pharmaceutics.

[28] Sobczak M. (2022). Enzyme-Responsive Hydrogels as Potential Drug Delivery Systems—State of Knowledge and Future Prospects. Int. J. Mol. Sci..

[29] Ghanbarzadeh B., Almasi H. (2013). Biodegradable Polymers. Biodegradation—Life of Science.

[30] Levalley P.J., Neelarapu R., Sutherland B.P., Dasgupta S., Kloxin C.J., Kloxin A.M. (2020). Photolabile Linkers: Exploiting Labile Bond Chemistry to Control Mode and Rate of Hydrogel Degradation and Protein Release. J. Am. Chem. Soc..

[31] Liu T., Bao B., Li Y., Lin Q., Zhu L. (2023). Photo-Responsive Polymers Based on ο-Nitrobenzyl Derivatives: From Structural Design to Applications. Prog. Polym. Sci..

[32] Meng F., Hennink W.E., Zhong Z. (2009). Reduction-Sensitive Polymers and Bioconjugates for Biomedical Applications. Biomaterials.

[33] Wang Q., Guan J., Wan J., Li Z. (2020). Disulfide Based Prodrugs for Cancer Therapy. RSC Adv..

[34] Brülisauer L., Gauthier M.A., Leroux J.C. (2014). Disulfide-Containing Parenteral Delivery Systems and Their Redox-Biological Fate. J. Control. Release.

[35] Mthembu S.N., Sharma A., Albericio F., de la Torre B.G. (2020). Breaking a Couple: Disulfide Reducing Agents. ChemBioChem.

[36] Condò I., Giannitelli S.M., Lo Presti D., Cortese B., Ursini O. (2024). Overview of Dynamic Bond Based Hydrogels for Reversible Adhesion Processes. Gels.

[37] Chang R., An H., Li X., Zhou R., Qin J., Tian Y., Deng K. (2017). Self-Healable Polymer Gels with Multi-Responsiveness of Gel-Sol-Gel Transition and Degradability. Polym. Chem..

[38] Kharkar P.M., Kiick K.L., Kloxin A.M. (2013). Designing Degradable Hydrogels for Orthogonal Control of Cell Microenvironments. Chem. Soc. Rev..

[39] Arif Z.U., Khalid M.Y., Sheikh M.F., Zolfagharian A., Bodaghi M. (2022). Biopolymeric Sustainable Materials and Their Emerging Applications. J. Environ. Chem. Eng..

[40] Vinchhi P., Rawal S.U., Patel M.M. (2020). Biodegradable Hydrogels. Drug Delivery Devices and Therapeutic Systems.

[41] Apriyanto A., Compart J., Fettke J. (2022). A Review of Starch, a Unique Biopolymer—Structure, Metabolism and in Planta Modifications. Plant Sci..

[42] Jiang T., Duan Q., Zhu J., Liu H., Yu L. (2020). Starch-Based Biodegradable Materials: Challenges and Opportunities. Adv. Ind. Eng. Polym. Res..

[43] Li M., Witt T., Xie F., Warren F.J., Halley P.J., Gilbert R.G. (2015). Biodegradation of Starch Films: The Roles of Molecular and Crystalline Structure. Carbohydr. Polym..

[44] Khlestkin V.K., Peltek S.E., Kolchanov N.A. (2018). Review of Direct Chemical and Biochemical Transformations of Starch. Carbohydr. Polym..

[45] Van Der Maarel M.J.E.C., Van Der Veen B., Uitdehaag J.C.M., Leemhuis H., Dijkhuizen L. (2002). Properties and Applications of Starch-Converting Enzymes of the a-Amylase Family. J. Biotechnol..

[46] Wang S., Copeland L. (2015). Effect of Acid Hydrolysis on Starch Structure and Functionality: A Review. Crit. Rev. Food Sci. Nutr..

[47] Sullivan W., Emeje M.O., Emeje M. (2020). Chemical Properties of Starch.

[48] Rutenberg M.W., Solarek D. (1984). Starch Derivatives: Production and Uses. Starch: Chemistry and Technology.

[49] Kondiah P.J., Choonara Y.E., Kondiah P.P.D., Marimuthu T., Kumar P., Du Toit L.C., Pillay V. (2016). A Review of Injectable Polymeric Hydrogel Systems for Application in Bone Tissue Engineering. Molecules.

[50] Zhang X., Jiang Y., Han L., Lu X. (2021). Biodegradable Polymer Hydrogel-Based Tissue Adhesives: A Review. Biosurface Biotribol..

[51] Houfani A.A., Anders N., Spiess A.C., Baldrian P., Benallaoua S. (2020). Insights from Enzymatic Degradation of Cellulose and Hemicellulose to Fermentable Sugars—A Review. Biomass Bioenergy.

[52] Mondal M.I.H., Mondal M.I.H. (2019). Polymers and Polymeric Composites: A Reference Series Cellulose-Based Superabsorbent Hydrogels.

[53] Entcheva E., Bien H., Yin L., Chung C.Y., Farrell M., Kostov Y. (2004). Functional Cardiac Cell Constructs on Cellulose-Based Scaffolding. Biomaterials.

[54] Erdal N.B., Hakkarainen M. (2022). Degradation of Cellulose Derivatives in Laboratory, Man-Made, and Natural Environments. Biomacromolecules.

[55] Toshikj E., Tarbuk A., Grgić K., Mangovska B., Jordanov I. (2019). Influence of Different Oxidizing Systems on Cellulose Oxidation Level: Introduced Groups versus Degradation Model. Cellulose.

[56] Pérez-Álvarez L., Ruiz-Rubio L., Vilas-Vilela J.L. (2018). Determining the Deacetylation Degree of Chitosan: Opportunities to Learn Instrumental Techniques. J. Chem. Educ..

[57] Dave U., Somanader E., Baharlouei P., Pham L., Rahman M.A. (2021). Applications of Chitin in Medical, Environmental, and Agricultural Industries. J. Mar. Sci. Eng..

[58] Joseph S.M., Krishnamoorthy S., Paranthaman R., Moses J.A., Anandharamakrishnan C. (2021). A Review on Source-Specific Chemistry, Functionality, and Applications of Chitin and Chitosan. Carbohydr. Polym. Technol. Appl..

[59] Unuofin J.O., Odeniyi O.A., Majengbasan O.S., Igwaran A., Moloantoa K.M.M., Khetsha Z.P., Iwarere S.A., Daramola M.O. (2024). Chitinases: Expanding the Boundaries of Knowledge beyond Routinized Chitin Degradation. Environ. Sci. Pollut. Res..

[60] Churklam W., Aunpad R. (2020). Enzymatic Characterization and Structure-Function Relationship of Two Chitinases, LmChiA and LmChiB, from Listeria Monocytogenes. Heliyon.

[61] Jiménez-Ortega E., Kidibule P.E., Fernández-Lobato M., Sanz-Aparicio J. (2021). Structural Inspection and Protein Motions Modelling of a Fungal Glycoside Hydrolase Family 18 Chitinase by Crystallography Depicts a Dynamic Enzymatic Mechanism. Comput. Struct. Biotechnol. J..

[62] Mushtaq A., Li L., Anitha A., Grøndahl L. (2021). Chitosan Nanomedicine in Cancer Therapy: Targeted Delivery and Cellular Uptake. Macromol. Biosci..

[63] Zaharoff D.A., Rogers C.J., Hance K.W., Schlom J., Greiner J.W. (2006). Chitosan solution enhances both humoral and cell-mediated immune responses to subcutaneous vaccination. Vaccine.

[64] Aranaz I., Alcántara A.R., Civera M.C., Arias C., Elorza B., Caballero A.H., Acosta N. (2021). Chitosan: An Overview of Its Properties and Applications. Polymers.

[65] Malerba M., Cerana R. (2020). Chitin- and Chitosan-Based Derivatives in Plant Protection against Biotic and Abiotic Stresses and in Recovery of Contaminated Soil and Water. Polysaccharides.

[66] Shariatinia Z., Jalali A.M. (2018). Chitosan-Based Hydrogels: Preparation, Properties and Applications. Int. J. Biol. Macromol..

[67] Kean T., Thanou M. (2010). Biodegradation, Biodistribution and Toxicity of Chitosan. Adv. Drug Deliv. Rev..

[68] Jennings J.A. (2016). Controlling Chitosan Degradation Properties In Vitro and In Vivo. Chitosan Based Biomaterials: Fundamentals: Volume 1.

[69] Boot R.G., Blommaart E.F.C., Swart E., Ghauharali-van der Vlugt K., Bijl N., Moe C., Place A., Aerts J.M.F.G. (2001). Identification of a Novel Acidic Mammalian Chitinase Distinct from Chitotriosidase. J. Biol. Chem..

[70] Eide K.B., Norberg A.L., Heggset E.B., Lindbom A.R., Varum K.M., Eijsink V.G.H., Sørlie M. (2012). Human Chitotriosidase-Catalyzed Hydrolysis of Chitosan. Biochemistry.

[71] Silva S.S., Oliveira J.M., Sá-Lima H., Sousa R.A., Mano J.F., Reis R.L. (2011). Polymers of Biological Origin. Comprehensive Biomaterials.

[72] Wardhani D.H., Ulya H.N., Hafizha M., Razani M.O.A. (2024). Effect of Temperatures on Modification of Alginate Hydrophobicity Using Dodecenyl Succinic Anhydride. E3S Web Conf..

[73] Chen C., Li X., Lu C., Zhou X., Chen L., Qiu C., Jin Z., Long J. (2024). Advances in Alginate Lyases and the Potential Application of Enzymatic Prepared Alginate Oligosaccharides: A Mini Review. Int. J. Biol. Macromol..

[74] Chesterman J., Zhang Z., Ortiz O., Goyal R., Kohn J. (2020). Biodegradable Polymers. Principles of Tissue Engineering.

[75] Guo X., Wang Y., Qin Y., Shen P., Peng Q. (2020). Structures, Properties and Application of Alginic Acid: A Review. Int. J. Biol. Macromol..

[76] Wang H., Chen X., Wen Y., Li D., Sun X., Liu Z., Yan H., Lin Q. (2022). A Study on the Correlation between the Oxidation Degree of Oxidized Sodium Alginate on Its Degradability and Gelation. Polymers.

[77] Zhu B., Yin H. (2015). Alginate Lyase: Review of Major Sources and Classification, Properties, Structure-Function Analysis and Applications. Bioengineered.

[78] Tønnesen H.H., Karlsen J. (2002). Alginate in Drug Delivery Systems. Drug Dev. Ind. Pharm..

[79] Pawar S.N., Edgar K.J. (2012). Alginate Derivatization: A Review of Chemistry, Properties and Applications. Biomaterials.

[80] Nouha K., Kumar R.S., Balasubramanian S., Tyagi R.D. (2018). Critical Review of EPS Production, Synthesis and Composition for Sludge Flocculation. J. Environ. Sci..

[81] Boran G., Mulvaney S.J., Regenstein J.M. (2010). Rheological Properties of Gelatin from Silver Carp Skin Compared to Commercially Available Gelatins from Different Sources. J. Food Sci..

[82] Chandra R. (1998). Biodegradable Polymers. Prog. Polym. Sci..

[83] Alipal J., Mohd Pu’ad N.A.S., Lee T.C., Nayan N.H.M., Sahari N., Basri H., Idris M.I., Abdullah H.Z. (2019). A Review of Gelatin: Properties, Sources, Process, Applications, and Commercialisation. Proceedings of the Materials Today: Proceedings.

[84] Lee K.Y., Mooney D.J. (2001). Hydrogels for Tissue Engineering. Chem. Rev..

[85] Van Den Bosch E., Gielens C. (2003). Gelatin Degradation at Elevated Temperature. Int. J. Biol. Macromol..

[86] Gray V.A., Cole E., Riva Toma J.M.D., Ghidorsi L., Guo J.H., Han J.H., Han F., Hosty C.T., Kochling J.D., Kraemer J. (2014). Use of Enzymes in the Dissolution Testing of Gelatin Capsules and Gelatin-Coated Tablets—Revisions to Dissolution <711> and Disintegration and Dissolution of Dietary Supplements <2040>. Dissolut Technol..

[87] Xiao H., Liu X., Feng Y., Zheng L., Zhao M., Huang M. (2022). Secretion of Collagenases by Saccharomyces Cerevisiae for Collagen Degradation. Biotechnol. Biofuels Bioprod..

[88] Guo B., Zhong Y., Chen X., Yu S., Bai J. (2023). 3D Printing of Electrically Conductive and Degradable Hydrogel for Epidermal Strain Sensor. Compos. Commun..

[89] Sagdic K., Fernández-Lavado E., Mariello M., Akouissi O., Lacour S.P. (2023). Hydrogels and Conductive Hydrogels for Implantable Bioelectronics. MRS Bull..

[90] Oladosu Y., Rafii M.Y., Arolu F., Chukwu S.C., Salisu M.A., Fagbohun I.K., Muftaudeen T.K., Swaray S., Haliru B.S. (2022). Superabsorbent Polymer Hydrogels for Sustainable Agriculture: A Review. Horticulturae.

[91] Dong L., Wang M., Wu J., Zhang C., Shi J., Oh K., Yao L., Zhu C., Morikawa H. (2023). Fully Biofriendly, Biodegradable and Recyclable Hydrogels Based on Covalent-like Hydrogen Bond Engineering towards Multimodal Transient Electronics. Chem. Eng. J..

[92] Pita-López M.L., Fletes-Vargas G., Espinosa-Andrews H., Rodríguez-Rodríguez R. (2021). Physically Cross-Linked Chitosan-Based Hydrogels for Tissue Engineering Applications: A State-of-the-Art Review. Eur. Polym. J..

[93] Slaughter B.V., Khurshid S.S., Fisher O.Z., Khademhosseini A., Peppas N.A. (2009). Hydrogels in Regenerative Medicine. Adv. Mater..

[94] Luo T., Tan B., Zhu L., Wang Y., Liao J. (2022). A Review on the Design of Hydrogels With Different Stiffness and Their Effects on Tissue Repair. Front. Bioeng. Biotechnol..

[95] Rami L., Malaise S., Delmond S., Fricain J.C., Siadous R., Schlaubitz S., Laurichesse E., Amédée J., Montembault A., David L. (2014). Physicochemical Modulation of Chitosan-Based Hydrogels Induces Different Biological Responses: Interest for Tissue Engineering. J. Biomed. Mater. Res. A.

[96] Sahranavard M., Zamanian A., Ghorbani F., Shahrezaee M.H. (2019). A Critical Review on Three Dimensional-Printed Chitosan Hydrogels for Development of Tissue Engineering. Bioprinting.

[97] Cao L., Werkmeister J.A., Wang J., Glattauer V., McLean K.M., Liu C. (2014). Bone Regeneration Using Photocrosslinked Hydrogel Incorporating RhBMP-2 Loaded 2-N, 6-O-Sulfated Chitosan Nanoparticles. Biomaterials.

[98] Miguel S.P., Ribeiro M.P., Brancal H., Coutinho P., Correia I.J. (2014). Thermoresponsive Chitosan-Agarose Hydrogel for Skin Regeneration. Carbohydr. Polym..

[99] Gnavi S., Barwig C., Freier T., Haastert-Talini K., Grothe C., Geuna S. (2013). The Use of Chitosan-Based Scaffolds to Enhance Regeneration in the Nervous System. International Review of Neurobiology.

[100] Tamimi M., Rajabi S., Pezeshki-Modaress M. (2020). Cardiac ECM/Chitosan/Alginate Ternary Scaffolds for Cardiac Tissue Engineering Application. Int. J. Biol. Macromol..

[101] Moreira M.S., Sarra G., Carvalho G.L., Gonçalves F., Caballero-Flores H.V., Pedroni A.C.F., Lascala C.A., Catalani L.H., Marques M.M. (2021). Physical and Biological Properties of a Chitosan Hydrogel Scaffold Associated to Photobiomodulation Therapy for Dental Pulp Regeneration: An in Vitro and in Vivo Study. Biomed. Res. Int..

[102] Seda Tığlı R., Karakeçili A., Gümüşderelioğlu M. (2007). In Vitro Characterization of Chitosan Scaffolds: Influence of Composition and Deacetylation Degree. J. Mater. Sci. Mater. Med..

[103] Niu X., Wei Y., Liu Q., Yang B., Ma N., Li Z., Zhao L., Chen W., Huang D. (2020). Silver-Loaded Microspheres Reinforced Chitosan Scaffolds for Skin Tissue Engineering. Eur. Polym. J..

[104] Zheng K., Feng G., Zhang J., Xing J., Huang D., Lian M., Zhang W., Wu W., Hu Y., Lu X. (2021). Basic Fibroblast Growth Factor Promotes Human Dental Pulp Stem Cells Cultured in 3D Porous Chitosan Scaffolds to Neural Differentiation. Int. J. Neurosci..

[105] Sadeghianmaryan A., Naghieh S., Alizadeh Sardroud H., Yazdanpanah Z., Afzal Soltani Y., Sernaglia J., Chen X. (2020). Extrusion-Based Printing of Chitosan Scaffolds and Their in Vitro Characterization for Cartilage Tissue Engineering. Int. J. Biol. Macromol..

[106] Goh C.Y., Lim S.S., Tshai K.Y., El Azab A.W.Z.Z., Loh H.S. (2019). Fabrication and in Vitro Biocompatibility of Sodium Tripolyphosphate-Crosslinked Chitosan–Hydroxyapatite Scaffolds for Bone Regeneration. J. Mater. Sci..

[107] Hu J., Hou Y., Park H., Choi B., Hou S., Chung A., Lee M. (2012). Visible Light Crosslinkable Chitosan Hydrogels for Tissue Engineering. Acta Biomater..

[108] Chang H.K., Yang D.H., Ha M.Y., Kim H.J., Kim C.H., Kim S.H., Choi J.W., Chun H.J. (2022). 3D Printing of Cell-Laden Visible Light Curable Glycol Chitosan Bioink for Bone Tissue Engineering. Carbohydr. Polym..

[109] Vo N.T.H., Huang L., Lemos H., Mellor A.L., Novakovic K. (2021). Genipin-Crosslinked Chitosan Hydrogels: Preliminary Evaluation of the in Vitro Biocompatibility and Biodegradation. J. Appl. Polym. Sci..

[110] Andrade del Olmo J., Pérez-Álvarez L., Sáez-Martínez V., Benito-Cid S., Ruiz-Rubio L., Pérez-González R., Vilas-Vilela J.L., Alonso J.M. (2022). Wound Healing and Antibacterial Chitosan-Genipin Hydrogels with Controlled Drug Delivery for Synergistic Anti-Inflammatory Activity. Int. J. Biol. Macromol..

[111] Farshidfar N., Iravani S., Varma R.S. (2023). Alginate-Based Biomaterials in Tissue Engineering and Regenerative Medicine. Mar. Drugs.

[112] Campbell K.T., Stilhano R.S., Silva E.A. (2018). Enzymatically Degradable Alginate Hydrogel Systems to Deliver Endothelial Progenitor Cells for Potential Revasculature Applications. Biomaterials.

[113] Zhang X., Li Y., Ma Z., He D., Li H. (2021). Modulating Degradation of Sodium Alginate/Bioglass Hydrogel for Improving Tissue Infiltration and Promoting Wound Healing. Bioact. Mater..

[114] Reakasame S., Boccaccini A.R. (2018). Oxidized Alginate-Based Hydrogels for Tissue Engineering Applications: A Review. Biomacromolecules.

[115] Bouhadir K.H., Lee K.Y., Alsberg E., Damm K.L., Anderson K.W., Mooney D.J. (2001). Degradation of Partially Oxidized Alginate and Its Potential Application for Tissue Engineering. Biotechnol. Prog..

[116] Jalali Kandeloos A., Bastani S., Mashayekhan S. (2023). Architecting Oxidized Alginate Methacrylate Hydrogels with Tunable Characteristics by Altering the Sequence of the Cross-Linking Steps, Methacrylation Reaction Time, and Polymer Concentration. J. Biomater. Appl..

[117] Sonaye S.Y., Ertugral E.G., Kothapalli C.R., Sikder P. (2022). Extrusion 3D (Bio)Printing of Alginate-Gelatin-Based Composite Scaffolds for Skeletal Muscle Tissue Engineering. Materials.

[118] Chen C., Xi Y., Weng Y. (2022). Recent Advances in Cellulose-Based Hydrogels for Tissue Engineering Applications. Polymers.

[119] Chimpibul W., Nakaji-Hirabayashi T., Yuan X., Matsumura K. (2020). Controlling the Degradation of Cellulose Scaffolds with Malaprade Oxidation for Tissue Engineering. J. Mater. Chem. B.

[120] Mirtaghavi A., Baldwin A., Tanideh N., Zarei M., Muthuraj R., Cao Y., Zhao G., Geng J., Jin H., Luo J. (2020). Crosslinked Porous Three-Dimensional Cellulose Nanofibers-Gelatine Biocomposite Scaffolds for Tissue Regeneration. Int. J. Biol. Macromol..

[121] Kaliampakou C., Lagopati N., Pavlatou E.A., Charitidis C.A. (2023). Alginate–Gelatin Hydrogel Scaffolds; An Optimization of Post-Printing Treatment for Enhanced Degradation and Swelling Behavior. Gels.

[122] Distler T., McDonald K., Heid S., Karakaya E., Detsch R., Boccaccini A.R. (2020). Ionically and Enzymatically Dual Cross-Linked Oxidized Alginate Gelatin Hydrogels with Tunable Stiffness and Degradation Behavior for Tissue Engineering. ACS Biomater. Sci. Eng..

[123] Shehzad A., Mukasheva F., Moazzam M., Sultanova D., Abdikhan B., Trifonov A., Akilbekova D. (2023). Dual-Crosslinking of Gelatin-Based Hydrogels: Promising Compositions for a 3D Printed Organotypic Bone Model. Bioengineering.

[124] Maturavongsadit P., Paravyan G., Shrivastava R., Benhabbour S.R. (2020). Thermo-/PH-Responsive Chitosan-Cellulose Nanocrystals Based Hydrogel with Tunable Mechanical Properties for Tissue Regeneration Applications. Materialia.

[125] Huang J., Jia Z., Liang Y., Huang Z., Rong Z., Xiong J., Wang D. (2019). Pulse Electromagnetic Fields Enhance the Repair of Rabbit Articular Cartilage Defects with Magnetic Nano-Hydrogel. RSC Adv..

[126] Goto R., Nishida E., Kobayashi S., Aino M., Ohno T., Iwamura Y., Kikuchi T., Hayashi J.I., Yamamoto G., Asakura M. (2021). Gelatin Methacryloyl–Riboflavin (Gelma–Rf) Hydrogels for Bone Regeneration. Int. J. Mol. Sci..

[127] El-Husseiny H.M., Mady E.A., El-Dakroury W.A., Zewail M.B., Noshy M., Abdelfatah A.M., Doghish A.S. (2022). Smart/Stimuli-Responsive Hydrogels: State-of-the-Art Platforms for Bone Tissue Engineering. Appl. Mater. Today.

[128] El-Husseiny H.M., Mady E.A., Hamabe L., Abugomaa A., Shimada K., Yoshida T., Tanaka T., Yokoi A., Elbadawy M., Tanaka R. (2022). Smart/Stimuli-Responsive Hydrogels: Cutting-Edge Platforms for Tissue Engineering and Other Biomedical Applications. Mater. Today Bio.

[129] Brandl F., Hammer N., Blunk T., Tessmar J., Goepferich A. (2010). Biodegradable Hydrogels for Time-Controlled Release of Tethered Peptides or Proteins. Biomacromolecules.

[130] Nambiar M., Schneider J.P. (2023). Cargo: Current and Potential Strategies. J. Peptide Sci..

[131] Peattie R.A., Pike D.B., Yu B., Cai S., Shu X.Z., Prestwich G.D., Firpo M.A., Fisher R.J. (2008). Effect of Gelatin on Heparin Regulation of Cytokine Release from Hyaluronan-Based Hydrogels. Drug Deliv..

[132] Chung H.J., Kim H.K., Yoon J.J., Park T.G. (2006). Heparin Immobilized Porous PLGA Microspheres for Angiogenic Growth Factor Delivery. Pharm. Res..

[133] Zhao Y., Zhang J., Wang X., Chen B., Xiao Z., Shi C., Wei Z., Hou X., Wang Q., Dai J. (2010). The Osteogenic Effect of Bone Morphogenetic Protein-2 on the Collagen Scaffold Conjugated with Antibodies. J. Control. Release.

[134] Lin C.C., Metters A.T. (2006). Enhanced Protein Delivery from Photopolymerized Hydrogels Using a Pseudospecific Metal Chelating Ligand. Pharm. Res..

[135] Liang Y., Zhao X., Ma P.X., Guo B., Du Y., Han X. (2019). PH-Responsive Injectable Hydrogels with Mucosal Adhesiveness Based on Chitosan-Grafted-Dihydrocaffeic Acid and Oxidized Pullulan for Localized Drug Delivery. J. Colloid. Interface Sci..

[136] Iglesias N., Galbis E., Valencia C., Díaz-Blanco M.J., Lacroix B., de-Paz M.V. (2020). Biodegradable Double Cross-Linked Chitosan Hydrogels for Drug Delivery: Impact of Chemistry on Rheological and Pharmacological Performance. Int. J. Biol. Macromol..

[137] Zhang Y., Wu B.M. (2023). Current Advances in Stimuli-Responsive Hydrogels as Smart Drug Delivery Carriers. Gels.

[138] Fan R., Cheng Y., Wang R., Zhang T., Zhang H., Li J., Song S., Zheng A. (2022). Thermosensitive Hydrogels and Advances in Their Application in Disease Therapy. Polymers.

[139] Preman N.K., Barki R.R., Vijayan A., Sanjeeva S.G., Johnson R.P. (2020). Recent Developments in Stimuli-Responsive Polymer Nanogels for Drug Delivery and Diagnostics: A Review. Eur. J. Pharm. Biopharm..

[140] Kumar N., Singh S., Sharma P., Kumar B. (2024). Single-, Dual-, and Multi-Stimuli-Responsive Nanogels for Biomedical Applications. Gels.

[141] Pertici V., Pin-Barre C., Rivera C., Pellegrino C., Laurin J., Gigmes D., Trimaille T. (2019). Degradable and Injectable Hydrogel for Drug Delivery in Soft Tissues. Biomacromolecules.

[142] Hussain AL-Mayahy M., Imad Hameed H. (2023). Hydrogels and Nanogels as a Promising Carrier for Drug Delivery. Hydrogels and Nanogels—Applications in Medicine.

[143] Zhang H., Zhai Y., Wang J., Zhai G. (2016). New Progress and Prospects: The Application of Nanogel in Drug Delivery. Mater. Sci. Eng. C.

[144] Myint S.S., Laomeephol C., Thamnium S., Chamni S., Luckanagul J.A. (2023). Hyaluronic Acid Nanogels: A Promising Platform for Therapeutic and Theranostic Applications. Pharmaceutics.

[145] Hang C., Zou Y., Zhong Y., Zhong Z., Meng F. (2017). NIR and UV-Responsive Degradable Hyaluronic Acid Nanogels for CD44-Targeted and Remotely Triggered Intracellular Doxorubicin Delivery. Colloids Surf. B Biointerfaces.

[146] Stefanello T.F., Couturaud B., Szarpak-Jankowska A., Fournier D., Louage B., Garcia F.P., Nakamura C.V., De Geest B.G., Woisel P., Van Der Sanden B. (2017). Coumarin-Containing Thermoresponsive Hyaluronic Acid-Based Nanogels as Delivery Systems for Anticancer Chemotherapy. Nanoscale.

[147] Pedrosa S.S., Pereira P., Correia A., Gama F.M. (2017). Targetability of Hyaluronic Acid Nanogel to Cancer Cells: In Vitro and in Vivo Studies. Eur. J. Pharm. Sci..

[148] Zhu Y., Wang X., Chen J., Zhang J., Meng F., Deng C., Cheng R., Feijen J., Zhong Z. (2016). Bioresponsive and Fluorescent Hyaluronic Acid-Iodixanol Nanogels for Targeted X-Ray Computed Tomography Imaging and Chemotherapy of Breast Tumors. J. Control. Release.

[149] Coninx S., Kalot G., Godard A., Bodio E., Goze C., Sancey L., Auzély-Velty R. (2022). Tailored Hyaluronic Acid-Based Nanogels as Theranostic Boron Delivery Systems for Boron Neutron Cancer Therapy. Int. J. Pharm. X.

[150] Lin Y., Li C., Liu A., Zhen X., Gao J., Wu W., Cai W., Jiang X. (2021). Responsive Hyaluronic Acid-Gold Cluster Hybrid Nanogel Theranostic Systems. Biomater. Sci..

[151] Hu Y., Hu S., Zhang S., Dong S., Hu J., Kang L., Yang X. (2021). A Double-Layer Hydrogel Based on Alginate-Carboxymethyl Cellulose and Synthetic Polymer as Sustained Drug Delivery System. Sci. Rep..

[152] Suhail M., Fang C.W., Chiu I.H., Khan A., Wu Y.C., Lin I.L., Tsai M.J., Wu P.C. (2023). Synthesis and Evaluation of Alginate-Based Nanogels as Sustained Drug Carriers for Caffeine. ACS Omega.

[153] Culebras M., Barrett A., Pishnamazi M., Walker G.M., Collins M.N. (2021). Wood-Derived Hydrogels as a Platform for Drug-Release Systems. ACS Sustain. Chem. Eng..

[154] Mehdi-Sefiani H., Granados-Carrera C.M., Romero A., Chicardi E., Domínguez-Robles J., Perez-Puyana V.M. (2024). Chitosan–Type-A-Gelatin Hydrogels Used as Potential Platforms in Tissue Engineering for Drug Delivery. Gels.

[155] Ogbeh G.O., Tsokar T.O., Salifu E. (2019). Optimization of Nutrients Requirements for Bioremediation of Spent-Engine Oil Contaminated Soils. Environ. Eng. Res..

[156] Mujtaba M., Sharif R., Ali Q., Rehman R., Khawar K.M. (2020). Biopolymer Based Nanofertilizers Applications in Abiotic Stress (Drought and Salinity) Control. Advances in Nano-Fertilizers and Nano-Pesticides in Agriculture: A Smart Delivery System for Crop Improvement.

[157] Silva A.C.Q., Silvestre A.J.D., Vilela C., Freire C.S.R. (2022). Natural Polymers-Based Materials: A Contribution to a Greener Future. Molecules.

[158] Shakiba S., Astete C.E., Paudel S., Sabliov C.M., Rodrigues D.F., Louie S.M. (2020). Emerging Investigator Series: Polymeric Nanocarriers for Agricultural Applications: Synthesis, Characterization, and Environmental and Biological Interactions. Environ. Sci. Nano.

[159] Otey F.H., Trimnell D., Westhoff R.P., Shasha B.S. (1984). Starch Matrix for Controlled Release of Urea Fertilizer. J. Agric. Food Chem..

[160] Teixeira M.A., Paterson W.J., Dum E.J., Li Q., Hunter B.K., Goosen M.F.A. (1990). Assessment of Chitosan Gels for the Controlled Release of Agrochemicals. Ind. Eng. Chem. Res..

[161] García M.C., Díez J.A., Vallejo A., García L., Cartagena M.C. (1996). Use of Kraft Pine Lignin in Controlled-Release Fertilizer Formulations. Ind. Eng. Chem. Res..

[162] Fertahi S., Ilsouk M., Zeroual Y., Oukarroum A., Barakat A. (2021). Recent Trends in Organic Coating Based on Biopolymers and Biomass for Controlled and Slow Release Fertilizers. J. Control. Release.

[163] Hou X., Pan Y., Xiao H., Liu J. (2019). Controlled Release of Agrochemicals Using PH and Redox Dual-Responsive Cellulose Nanogels. J. Agric. Food Chem..

[164] Dhiman A., Sharma A.K., Bhardwaj D., Agrawal G. (2023). Biodegradable Dual Stimuli Responsive Alginate Based Microgels for Controlled Agrochemicals Release and Soil Remediation. Int. J. Biol. Macromol..

[165] Thombare N., Mishra S., Siddiqui M.Z., Jha U., Singh D., Mahajan G.R. (2018). Design and Development of Guar Gum Based Novel, Superabsorbent and Moisture Retaining Hydrogels for Agricultural Applications. Carbohydr. Polym..

[166] Tanan W., Panichpakdee J., Saengsuwan S. (2019). Novel Biodegradable Hydrogel Based on Natural Polymers: Synthesis, Characterization, Swelling/Reswelling and Biodegradability. Eur. Polym. J..

[167] Gabriel G.R., Joshua G.B., Ashley Y.C., Jake N.P., Terence T. (2020). Synthesis and Characterization of Sodium Carboxymethyl Cellulose/Sodium Alginate/Hydroxypropyl Cellulose Hydrogel for Agricultural Water Storage and Controlled Nutrient Release. Proceedings of the Solid State Phenomena.

[168] Passauer L., Hallas T., Bäucker E., Ciesielski G., Lebioda S., Hamer U. (2015). Biodegradation of Hydrogels from Oxyethylated Lignins in Model Soils. ACS Sustain. Chem. Eng..

[169] Sharma G., Saharan V., Pal A., Sharma S.S. (2024). Chitosan Nanofertilizer to Strengthen Sink Strength and Provide Resistance against PFSR (Post Flowering Stalk Rot) Disease in Maize. Biocatal. Agric. Biotechnol..

[170] Song B., Liang H., Sun R., Peng P., Jiang Y., She D. (2020). Hydrogel Synthesis Based on Lignin/Sodium Alginate and Application in Agriculture. Int. J. Biol. Macromol..

